# Landscape quantifies the intermediate state and transition dynamics in ecological networks

**DOI:** 10.1371/journal.pcbi.1011766

**Published:** 2024-01-05

**Authors:** Jinchao Lv, Jin Wang, Chunhe Li

**Affiliations:** 1 Institute of Science and Technology for Brain-Inspired Intelligence, Fudan University, Shanghai, China; 2 Shanghai Center for Mathematical Sciences, Fudan University, Shanghai, China; 3 Department of Chemistry and of Physics and Astronomy, State University of New York, Stony Brook, New York, United States of America; 4 School of Mathematical Sciences and MOE Frontiers Center for Brain Science, Fudan University, Shanghai, China; Dartmouth College, UNITED STATES

## Abstract

Understanding the ecological mechanisms associated with the collapse and restoration is especially critical in promoting harmonious coexistence between humans and nature. So far, it remains challenging to elucidate the mechanisms of stochastic dynamical transitions for ecological systems. Using an example of plant-pollinator network, we quantified the energy landscape of ecological system. The landscape displays multiple attractors characterizing the high, low and intermediate abundance stable states. Interestingly, we detected the intermediate states under pollinator decline, and demonstrated the indispensable role of the intermediate state in state transitions. From the landscape, we define the barrier height (BH) as a global quantity to evaluate the transition feasibility. We propose that the BH can serve as a new early-warning signal (EWS) for upcoming catastrophic breakdown, which provides an earlier and more accurate warning signal than traditional metrics based on time series. Our results promote developing better management strategies to achieve environmental sustainability.

## Author summary

Exploring the evolutionary characteristics of complex ecological networks is an important issue. Here, using a plant-pollinator network as an example, we investigate the stochastic dynamical mechanism of the ecological system. The multistable energy landscape characterizes high, low, and intermediate abundance stable states. Interestingly, we observe the intermediate states during pollinator decline and reveal their vital role in state transitions. The barrier height calculated from landscape provides a global measure to quantify the feasibility of transitions. We propose that the barrier height can serve as a new early warning signal (EWS) for anticipating catastrophic breakdown, which is more accurate and prompt than traditional metrics based on time-series. Our findings highlight the need for improved management strategies to achieve environmental sustainability.

## Introduction

Current ecosystems are under grave and unprecedented threat, due to combined effects of worse external conditions and weaker internal interactions [1, 2]. The damage can result in the extinction of some species, which has particularly negative impacts on both biodiversity and stability [3, 4]. The sudden collapse, also known as a regime shift phenomenon, can occur unpredictably [5]. It is an irreversible changeover in relation to the survival of all species [6, 7]. To describe the process of evolution, deterministic models have proved to be valid tools [8, 9], while the stochasticity is also vital, since the noise plays an essential part in transitions between healthy state and degraded state [10, 11]. However, how to interrogate the stochastic transition dynamics for a complex system has been a challenging problem. The concept of energy landscape provides a way to quantify stochastic dynamics and relative stability [12, 13]. It brings us with valuable opportunities for quantitative calculation, global representation and discovery of the states unnoticed in the deterministic model [14], and is extensively utilized in gene regulatory networks [15, 16], computational neuroscience [17, 18] and ecological systems [14, 19].

An important example in ecosystems is plant-pollinator mutualistic network, in which pollinators play significant functional roles in maintaining biological diversity and enhancing crucial pollination services to plants. Field studies have shown widespread and accelerating losses in pollinator richness and abundance [20, 21], as well as parallel decay in linked plants [22]. Underlying drivers include pesticide use, alien species, land loss and climatic change [23–26]. Several research efforts reveal the fact that most pollinators are prone to faster extinctions in comparison to plants [27, 28]. Pollinator decline deeply disrupts reproductive behaviors of plant populations [29, 30], which in turn further reduce pollinator densities as a result of decreased pollen quality and quantity [31]. This positive feedback causes a vicious circle of attenuation in many species. To model the uncertain extinction risk in pollinators, a common operation is to randomly select and remove a certain proportion of them in observational networks [8, 32, 33]. What’s more, two frequently studied states, the high-abundance stable state and the alternative low-abundance stable state, are truly widespread and coexisting with each other owing to the presence of a hysteresis loop in both low-dimensional and high-dimensional models [8, 10, 32]. A growing body of literature recognizes the importance of bistability, including prediction of tipping points [8, 33], managing strategies of influential pollinators [32, 34], and resilient network structures [35, 36]. Nevertheless, it remains challenging to quantify the stochastic transition dynamics for ecological networks, especially when multistability phenomenon emerges. Besides, previous studies were mostly concentrated on low-dimensional mean-field models [8, 14, 33], where information may be lost, such as the heterogeneity in species interactions.

In this work, to quantify the effects of perturbation and clarify the mechanisms of evolution, we studied the stochastic transition dynamics of ecological networks using the landscape and transition path theory, by taking the plant-pollinator mutualistic network as an example. Interestingly, we identified new intermediate states in the context of pollinator loss. Next, an in-depth analysis was performed for the network that exhibits tristability (three stable states). We calculated the kinetic transition paths between coexisting attractors, which provide the order information of species extinction and recovery. We found the irreversible transitions caused by the probabilistic flux that measures the extent of detailed balance broken in non-equilibrium systems. More importantly, by comparing the direct and indirect paths, we proposed that the intermediate state can be treated as an indicator of impending sudden collapse. As an extension of previous dimension reduction approach for landscape [37], we projected the high-dimensional landscape onto new coordinates inspired by hierarchical principal component analysis [38, 39], which is more applicable to this bipartite system. Calculated from the landscape topography, the barrier height (BH) measures relative stability of each attractor. Under conditions of environmental decline, the BH demonstrates similar patterns with transition action and mean first passage time (MFPT), both of which directly portray the difficulty of state transitions. Remarkably, we found that the BH provides a new early-warning signal (EWS) of final collapse, which performs significantly earlier and more accurate than traditional metrics derived from time series, including autocorrelation and variance. We also conducted global sensitivity analysis and interpreted ecological management strategies. To mitigate the collapse process, we only need to protect the pollinators surviving in the intermediate state. However, it is not far enough in terms of full recovery. Our model predicts that one should produce more favorable conditions to achieve recovery to the high state. The conclusions regarding BH as EWS and ecological strategies hold for multiple networks by considering the heterogeneity of the reciprocal interaction. We also demonstrated that a multidimensional model is necessary for the appearance of the intermediate states. Overall, our approach provides a general framework for understanding stochastic transition dynamics and sustainability in ecological systems.

## Results

### Appearance of multistability in the plant-pollinator network

**Fig 1:**
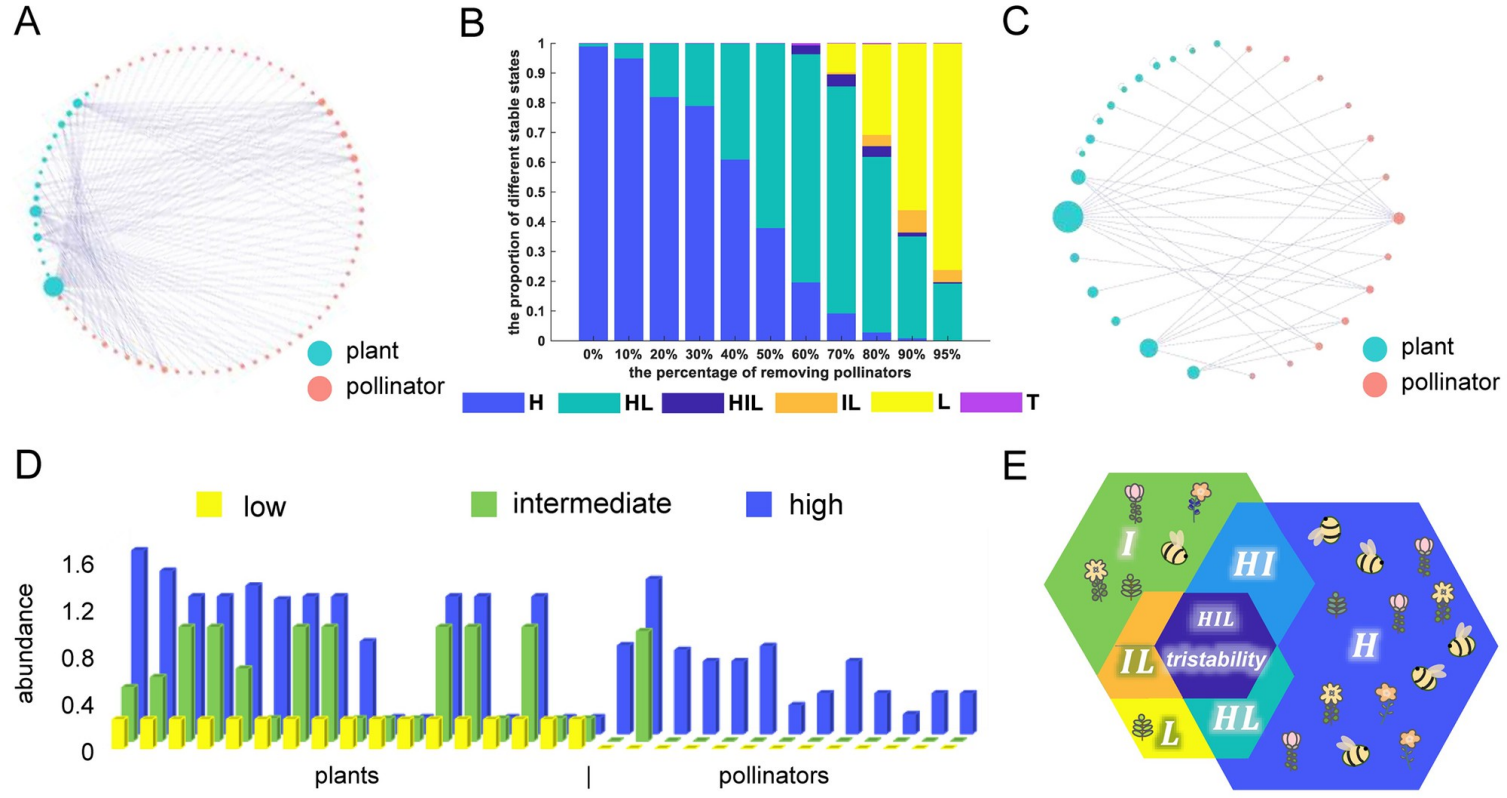
Multistability in the plant-pollinator network. (A) Raw network structure of an empirical mutualistic network from actual observation data [40]. (B) The proportion of different stable states (y-axis) with random removal of a certain percentage of pollinators (x-axis). Total 1000 sub-networks are simulated in each column except for the first (0% represents the original network). (C) A typical plant-pollinator network after removing 80% of pollinators, in which tristability occurs. (D) The abundance of 17 plant species and 13 pollinator species (indicated by each column) in low, intermediate and high stable states using default parameters. The default values are set as follows: $\alpha_{i}^{\left(P\right)}=\alpha_{j}^{\left(A\right)}=0.3$, $\beta_{ii}^{\left(P\right)}=\beta_{jj}^{\left(A\right)}=1$, $\beta_{ik}^{\left(P\right)}=\beta_{jm}^{\left(A\right)}=0.01\left(i\neq k,j\neq m\right)$, *κ* = 1.07, *γ*_0_ = 1, *δ* = 0.5, *h* = 0.2, *μ*^(*P*)^ = *μ*^(*A*)^ = 0.001. (E) Illustration of seven possible stable states, including monostable state, bistability and tristability. H: high state, HI: high-intermediate bistability, HL: high-low bistability, HIL: high-intermediate-low tristability, IL: intermediate-low bistability, L: low state, T: tetrastability.

We obtained plant–pollinator mutualistic networks from the Web of Life database (www.Web-of-Life.es) and focused on a widely studied network consisting of 17 plants and 61 pollinators from Hickling, Norfolk, UK [40], where we have already known the network structure and parameter range for bistability (Fig 1A). Based on generalized Lotka-Volterra dynamics, the *i*^*th*^ plant *P*_*i*_ and the *j*^*th*^ pollinator *A*_*j*_ conform to the following non-linear ordinary differential equations (ODEs) respectively [8, 35]:

$$\begin{array}{c} \begin{array}{c} \begin{array}{cc} \frac{dP_{i}}{dt}= & \alpha_{i}^{\left(P\right)}P_{i}-\beta_{ii}^{\left(P\right)}P_{i}^{2}-\sum_{k=1,k\neq i}^{N_{P}}\beta_{ik}^{\left(P\right)}P_{i}P_{k}+\frac{P_{i}\sum_{l=1}^{N_{A}}\gamma_{il}^{\left(A\right)}A_{l}}{1+h\sum_{l=1}^{N_{A}}\gamma_{il}^{\left(A\right)}A_{l}}+\mu^{\left(P\right)},\\ \frac{dA_{j}}{dt}= & \alpha_{j}^{\left(A\right)}A_{j}-\kappa A_{j}-\beta_{jj}^{\left(A\right)}A_{j}^{2}-\sum_{m=1,m\neq j}^{N_{A}}\beta_{jm}^{\left(A\right)}A_{j}A_{m}+\frac{A_{j}\sum_{n=1}^{N_{P}}\gamma_{jn}^{\left(P\right)}P_{n}}{1+h\sum_{n=1}^{N_{P}}\gamma_{jn}^{\left(P\right)}P_{n}}+\mu^{\left(A\right)}, \end{array} \end{array} \end{array}$$ (1)

where *α*_*i*_ represents the intrinsic growth rate, *β*_*ii*_ and *β*_*ik*_ represent the rate for intraspecific competitions and interspecific competitions, respectively. *κ* is the average decay rate of pollinators affected by environmental deterioration. *h* denotes the half-saturation constant and the immigration rate is denoted by *μ*. The reciprocal strength $\gamma_{il}^{\left(A\right)}$ is defined as $\gamma_{il}^{\left(A\right)}=\gamma_{0}\varepsilon_{il}/{\left[g_{i}^{\left(P\right)}\right]}^{\delta }$, among which *γ*_0_ is the per capita strength, *ε*_*il*_ indicates whether a link exists between the *i*^*th*^ plant and the *l*^*th*^ pollinator, $g_{i}^{\left(P\right)}$ is the degree of the *i*^*th*^ plant and *δ* is a trade-off parameter (see Methods for details).

Meanwhile, to model how the progressively worsening conditions disturb the system, we carried out common operations for random removal of a certain percentage of pollinators, as well as their links to plants [8, 32, 33]. Hence, for each removal percentage, we randomly generated 1000 sub-networks. Then we obtained stable state results by simulations of ODEs, and showed the frequency distributions of different types of stable states in each case (Fig 1B). It reveals a marked fall in the proportion of high monostable (single stable) state while rise in that of low monostable state with soaring removal rate. The percentage of the high-low bistable state increases firstly and decreases afterward, predominating in removing 50% to 80% of pollinators. What surprises us is the appearance of multistability, like tristability and tetrastability that involves one and two intermediate states respectively. The intermediate states are identified in some networks from which more than half of pollinators are removed (Fig 1B). The characteristics of the intermediate states are that less than half of (or even only one) pollinator species remain alive and plants are generally present in moderate abundance. This phenomenon derives from the strong nonlinearity induced by the mutualistic interactions in dynamics, endogenous positive plant-pollinator feedbacks, as well as underlying topological structures of networks.

Without loss of generality, we used a sub-network exhibiting tristability as an example, and performed further analysis. It is composed of 17 plants and 13 pollinators, generated by removing 80% of pollinator nodes (Fig 1C and Table A in S1 Text). Importantly, we propose that this plant-pollinator system can generate tristable state (three stable states), that is, the high, low and intermediate states, and the naming for states is based on the ranking of species abundance. For the high state, the abundance of both plants and pollinators is ample. For the low state, all pollinators go extinct and plants are also rare (Fig 1D). For the intermediate state, most plants are still alive but with an intermediate level of abundance, and the most generalist pollinator (*Bombus pascuorum*) is the only surviving pollinator (Fig 1D). Considering the coexistence of stable states, seven possible stable state scenarios are displayed (Fig 1E). Each scenario can be found in this system, and varying different parameters results in the phase change. Noise can also induce a phase transition of the system, and thus affect species abundance (see Section A and Fig A in S1 Text for details). In particular, the appearance of the intermediate state allows the system to exhibit distinct patterns that were rarely reported previously.

### Multistable landscape and transition path reveal the role of intermediate state

**Fig 2:**
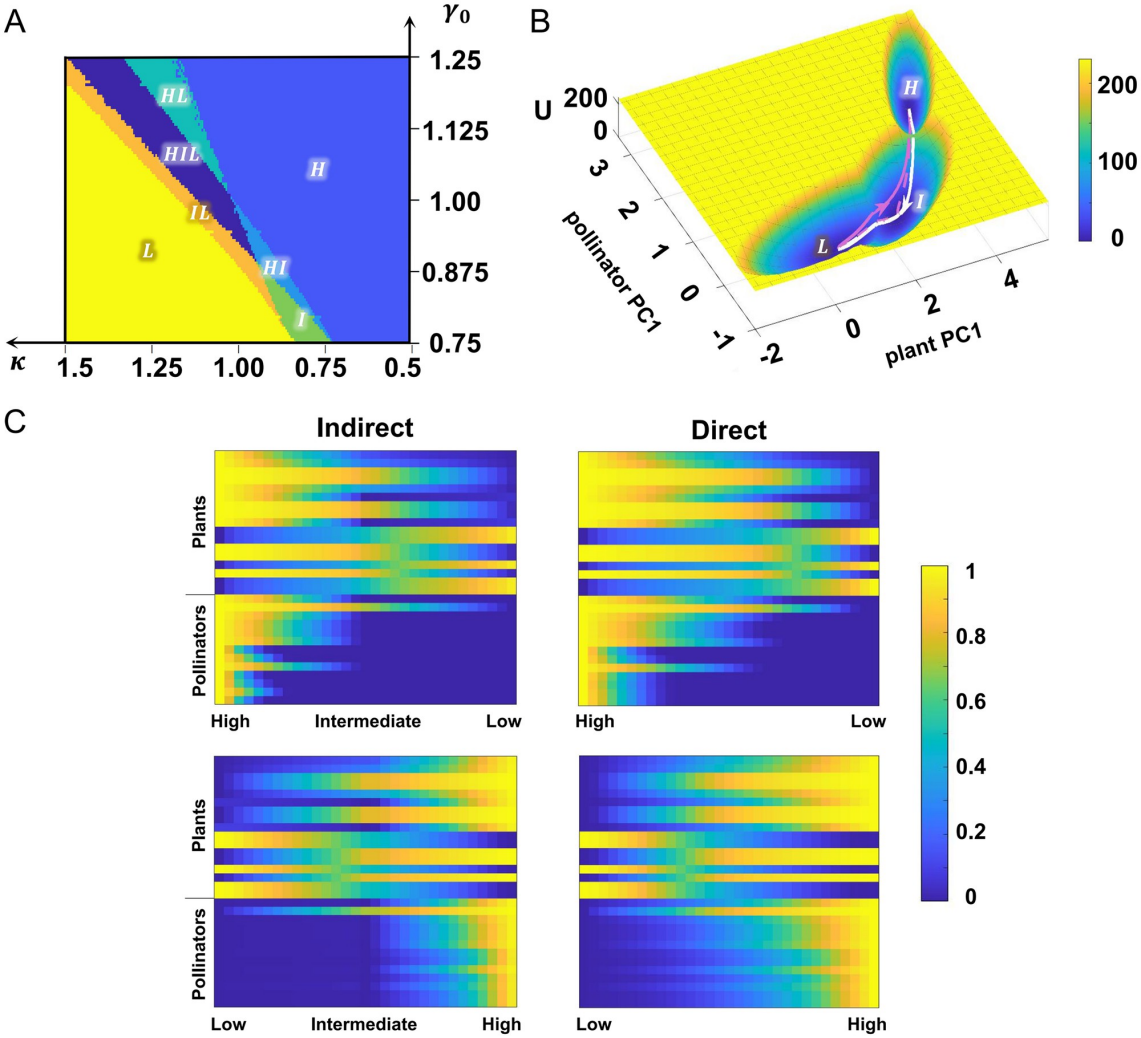
Multistable landscape and transition path reveal the role of intermediate state. (A) The phase diagram under parameter variation (*κ*=0.5∼1.5, *γ*_0_=0.75∼1.25). The same notations apply as in Fig 1E. I: intermediate state. (B) Landscape and transition paths after projection onto new coordinates in the tristable system (*κ* = 1.1, *γ*_0_ = 1). The white lines indicate the collapse process while the magenta lines represent the recovery process. The solid and dashed lines correspond to direct and indirect transition paths, respectively. (C) Multidimensional transition paths between different attractors after normalization, where two left pictures are indirect paths (through the intermediate state) from high to low state (top) and low to high state (bottom); conversely, the two on the right are direct paths. Each row represents one of 30 species in the network and the upper 17 rows are plants while the lower 13 rows are pollinators.

To explore the impacts of two critical parameters (*κ* and *γ*_0_) on the system dynamics, we plotted the phase diagram over a broad range of parameters: *κ*=0.5∼1.5, *γ*_0_=0.75∼1.25 (Fig 2A and see Section B in S1 Text for details). Intuitively, large *κ* (decay rate of pollinators) leads to population declines but large *γ*_0_ (per capita mutualistic strength) enhances the abundance of both plants and pollinators. We discovered that the system exhibits the intermediate monostable state at smaller *κ* and smaller *γ*_0_, but tristable state when *κ* and *γ*_0_ both increase along the diagonal (Fig 2A). This is reasonable, since larger *κ* and larger *γ*_0_ promote the generation of the low state and high state respectively, and their simultaneous increase promotes the appearance of multiple stable states.

To study the stochastic transition dynamics involved in tristable state, we quantified the energy landscape of the plant-pollinator network in the tristability region. The landscape takes into account not only the stability from a global perspective, but also stochastic effects including noise-induced transitions. Additionally, through a dimension reduction approach for landscape, we are able to display the high-dimensional landscape in reduced coordinates, with good interpretability (see Methods and Section C in S1 Text for details). The potential energy *U* is quantified through: *U*(***x***) = −log(*p*_*ss*_(***x***)), where *p*_*ss*_(***x***) is the stationary probability density (see Methods for details). Here an attractor represents stable-state abundance of each species, and the state transition process is viewed as the barrier-crossing process.

To explore the dynamical switching processes, based on the principle of least action, we calculated transition paths which reflect the most likely pathway from one attractor to another, and projected them onto the reduced dimensions (Fig 2B). Here we added the physical constraint that the abundance of each species must be non-negative within the paths. We roughly observed that the indirect and direct paths are exactly alike during switching process from high to low state, that is, the system tends to proceed through the intermediate state before final collapse. The multidimensional transition paths confirm this observation as well. The abundance of each species is linearly normalized to the range [0, 1], where 0 and 1 correspond to its respective minimum and maximum (Fig 2C). We evaluated the similarity between indirect and direct paths using the squared Euclidean distance in high-dimensional space. The path distance (PD) is defined as $\text{PD}=\sum_{i=1}^{L}{{∥y_{i}^{\left(\text{direct}\right)}-y_{i}^{\left(\text{indirect}\right)}∥}_{2}}^{2}$, where *L* is the number of nodes in the path, and we chose *L* = 20 (see Fig B in S1 Text for how *L* is chosen). $y_{i}^{\left(\text{direct}\right)}$ and $y_{i}^{\left(\text{indirect}\right)}$ represent each species abundance at the *i*^*th*^ node in direct and indirect paths respectively. We found two paths from high to low state are closer (PD_high→low_ = 8.5811) than those from low to high state (PD_low→high_ = 15.6319). Previous studies have demonstrated that ecosystems abruptly disrupt from a healthy state to an alternative state at a tipping point [5, 8, 35]. Here the existence of the intermediate state provides a new perspective on early indicators for critical transitions in ecology. Transition from the high state to the intermediate state signifies that a collapse is in progress, which can remind people to implement the associated environmental strategy to mitigate the collapse.

We also noticed that some plants have relatively higher abundance in the low state while lower abundance in the high state (Fig 2C). We examined that they have no connections with pollinators, thus the interspecific competitions become dominant and lead to such phenomenon. In addition, it is apparent that the attenuation starts at pollinators, identified both in projected and multidimensional paths (Fig 2B and 2C). Therefore, pollinators are regarded as the cause of collapse transition, which are consistent with previous studies [30, 41, 42]. It suggests that effective interventions for pollinators could inhibit ecosystem breakdown to a certain extent. But for recovery, it first occurs in plants, which is also corroborated in simulated stochastic trajectories (Fig A in S1 Text). With regard to direct path, other pollinators are recovered earlier and the system moves away from the low state faster than that for indirect path (Fig 2C). The understanding about possible causal mechanisms provides key insights into ecological strategies.

In addition, we also considered the scenario of colored noise (including red noise and blue noise) and used Langevin simulations to construct the energy landscape (Fig C in S1 Text). We found that the red noise promotes the occurrence of state transition behavior due to the positive correlation of noise between adjacent time points. When there is a transition trend in a certain direction, the noise at the next time point intensifies this tendency. Conversely, blue noise has the opposite effect.

### Landscape changes with parameter variation

While previous studies mostly focused on simulation of individual trajectories [10, 34, 43], the landscape picture provides a global description for attractor stability and state transitions. We can quantify the landscape through truncated moment equation approach (see Methods for details). For the high-dimensional landscape, how to visualize it is a challenging problem. We have developed different ways to deal with it.

We developed a dynamical model-based dimension reduction approach for the landscape [37]. However, the top two principal components (PCs) selected as coordinates do not display good biological meanings (see Fig D in S1 Text for details), which hardly visualize the changes of abundance in plants and pollinators. Also, the reduced landscape shows instability as shown from changes in weights and corresponding attractor positions after a small perturbation of parameter *κ* (see Figs D and E in S1 Text for details). Here, for the bipartite system, we proposed a new dimension reduction approach for the landscape (see Methods and Section C in S1 Text for details). Inspired by hierarchical principal component analysis [38, 39], we separately selected the first PC (PC1) of plant covariance matrix and PC1 of pollinator covariance matrix for projection, which endows the coordinates with good biological meanings and interpretability. Meanwhile, we also demonstrated the robustness of the coordinates under parameter perturbations (see Figs D and E in S1 Text for details), allowing us to casually determine the reference coordinates to ensure comparability.

**Fig 3:**
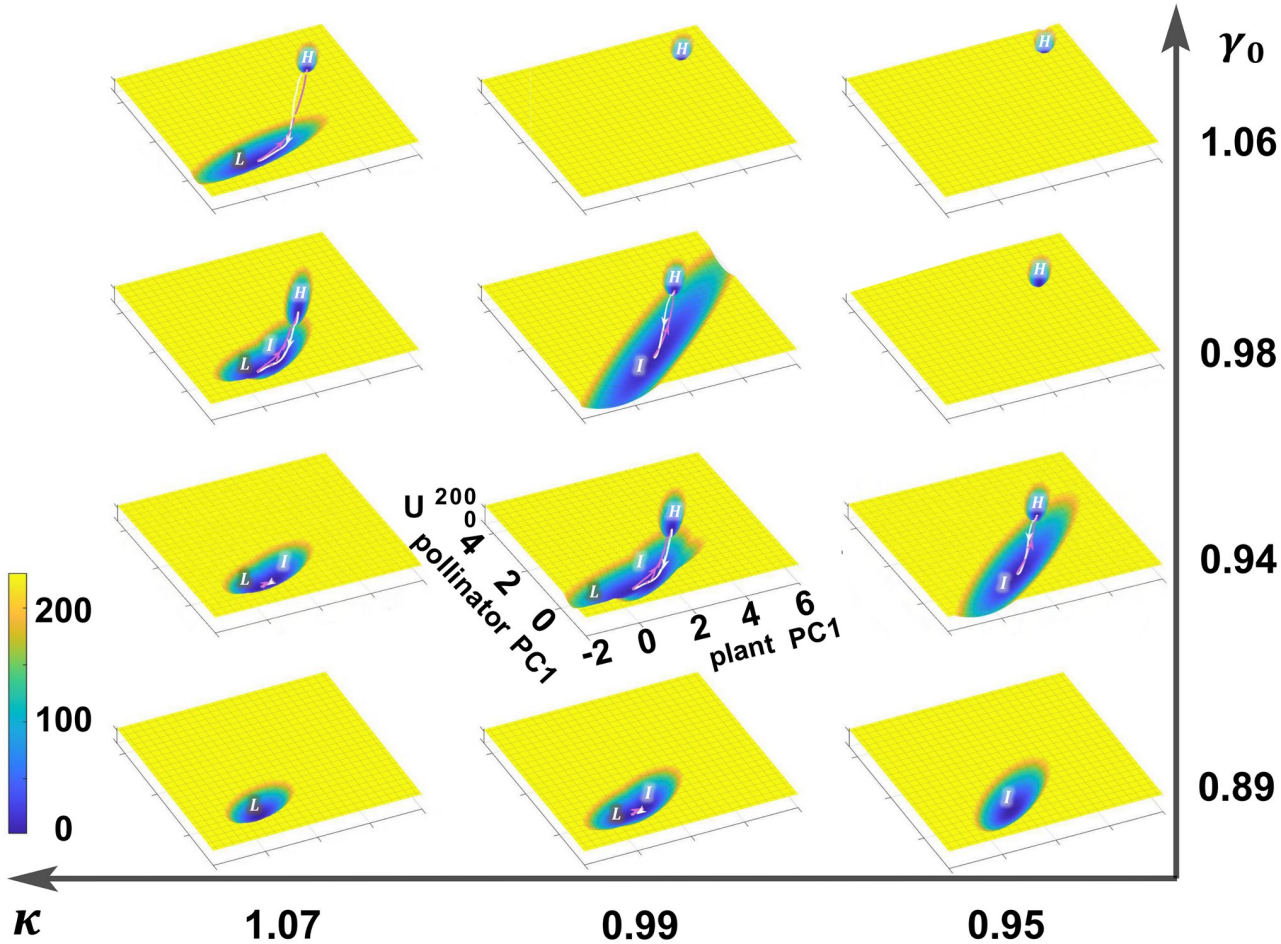
Landscape changes with parameters *κ* and *γ*_0_. All pictures share the coordinates of *κ*=0.99 and *γ*_0_=0.94, which are uniquely labelled for simplicity. For each landscape, small and large values of plant PC1 / pollinator PC1 correspond to low state (lower left corner) and high state (upper right corner) respectively, and moderate value is related to the intermediate state. Blue color indicates the basin of attraction corresponding lower potential energy or higher probability, while yellow color indicates high potential energy or lower probability. The transition paths between different stable states are also displayed, as in Fig 2B.

With the aim of showing every possible stable state scenario, we displayed a series of landscapes on 12 pairs of parameters which include *κ*=0.95, 0.99, 1.07 and *γ*_0_=0.89, 0.94, 0.98, 1.06, respectively (Fig 3). To ensure mutual comparability, all coordinates are taken from those calculated under *κ*=0.99, *γ*_0_=0.94. In the framework of Waddington, the abundance of all species can be regarded as a ball residing on the surface of landscape, always going downhill in the lack of outside interventions. The initial condition affects the stable state position when there are multiple basins. External forces or noise can trigger transitions between multiple states, but has little effect on monostable case. As *κ* increases or *γ*_0_ decreases, the system progressively transits from high state to low state, probably via high-intermediate bistable state, high-low bistable state, intermediate monostable state, high-intermediate-low tristable state, and intermediate-low bistable state (Fig 3). Different ways to adjust parameters influence the process of collapse, but the intermediate state always appears in the process. For full restoration, the environmental condition required is weaker when the mutualistic strength is larger, and vice versa. We also noticed the intermediate state is close to the low state in the landscape, because the system at such a transition state is facing a precarious situation when only one pollinator remains alive.

### Quantification of barrier height, transition action and mean first passage time

**Fig 4:**
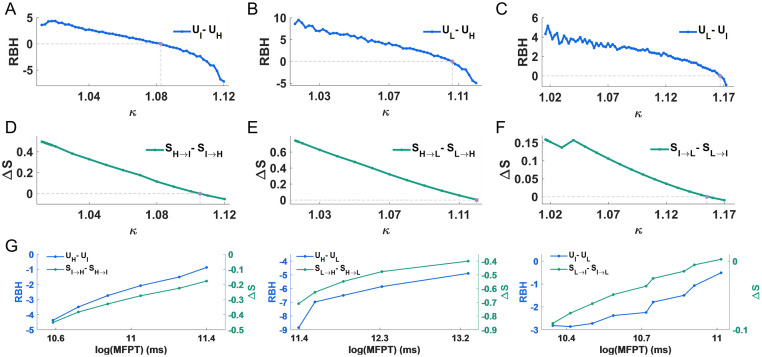
Consistency among barrier height, transition action and MFPT. (A-C) A line graph of RBH between stable-state pairs with varying *κ* (A: intermediate and high state; B: low and high state; C: low and intermediate state). We also label the turning point (purple point), where two stable states have the same potential energy value (RBH = 0). (D-F) The difference for the minimum actions between the forward and backward transitions (ΔS) vary with *κ* (D: intermediate and high state; E: low and high state; F: low and intermediate state). (G) Approximate linear relationships between RBH and log(MFPT) (blue line), as well as between ΔS and log(MFPT) (green line).

Ecological resilience is concerned with the amount of perturbation that a system can tolerate without shifting to an alternative state [5]. Here the problem corresponding to resilience is to precisely quantify the difficulty of transition from an attractor to another. We concentrated on the impact of parameter perturbation, and demonstrated the continuing change across stable states with environmental degradation. To measure the relative stability of attractor ***x***^*i*^ and attractor ***x***^*j*^, we firstly define the barrier height (BH) as BH_*si*_ = *U*_saddle_ − *U*_*i*_, i.e., the potential energy difference between the saddle point and local minimum (corresponding to ***x***^*i*^). Then, the relative barrier height (RBH) is defined as RBH_*ij*_ = BH_*sj*_ − BH_*si*_, which quantifies the relative transition feasibility between state ***x***^*i*^ and state ***x***^*j*^. In the bistable or tristable region, we reported the variations of RBH between pairs of stable states in the case of changing *κ* (Fig 4A, 4B and 4C). What stands out is the general pattern of dramatic decline which suggests the rapid rise in potential energy of high state. In other words, the high state is increasingly unstable. Nevertheless, the low state shows an opposite trend, i.e., becoming more and more stable. It is interesting that the potential energy of intermediate state first decreases and then increases as *κ* soars. Then we deduced that the change can be broadly divided into two stages. As for the two-stage collapse process, the system first transits from the high state to the intermediate state, until the high state disappears at around *κ* ≤ 1.12 (Fig 4A and 4B). Afterwards (approximately 1.12 ≤ *κ* ≤ 1.17), the intermediate state progressively shifts toward the low state, but the former keeps more stable until approaching the tipping point (Fig 4C). The entire process further confirms the sudden collapse of ecosystem and supports the real existence of intermediate state as well as the hypothesis of going through it.

Moreover, the minimum transition action, corresponding to the most likely transition path between attractors, is a direct representation for transition feasibility. It tends to be more computationally expensive since its calculation is based on original multidimensional system (see Methods for details). We showed the differences of the transition actions between the forward and backward paths (Δ*S*) (Fig 4D, 4E and 4F), which also measure the relative stability of attractors. Then we found that, in the early first stage (around *κ* ≤ 1.11), it is easier to transit from intermediate to high state, and in the late first stage (around 1.11 ≤ *κ* ≤ 1.12), the reverse is more likely to occur (Fig 4D). Also, transition from high to low state does not occur easily in the first stage (Fig 4E). In the second stage, it is not until close to the critical point that intermediate to low transition is easier than the opposite direction (around 1.16 ≤ *κ* ≤ 1.17) (Fig 4F). The results consistent with those of RBH reflect the fact that the transition from high-potential state to low-potential state is easier than the reverse (see Fig F in S1 Text for details). Additionally, we marked the turning points (defined as RBH = 0 and Δ*S* = 0 respectively), and their corresponding *κ* values are very similar. They further illustrate that the RBH holds sufficient information about the original system, and it can quantitatively elucidate the change.

Besides, the mean first passage time (MFPT) provides another indicator of state transitions. It can be obtained from multidimensional trajectory simulation of noisy species abundance. We applied the corresponding Langevin equations under fixed parameters (see Methods and Section D in S1 Text for details). Based on Euler-Maruyama method, we recorded each first passage time from one attractor to the other. The average was taken to approximate the MFPT (see Fig F in S1 Text for details). With different *κ*, we selected the direction where the transition is likely to occur, and all clarified the approximate linear relationships between RBH and the logarithm of MFPT (log(MFPT)), as well as Δ*S* and log(MFPT) (Fig 4G and see Fig F in S1 Text for details). The above three quantitative metrics give us the opportunity to properly describe the dynamics in system change.

### Barrier height as a new EWS to predict collapse

**Fig 5:**
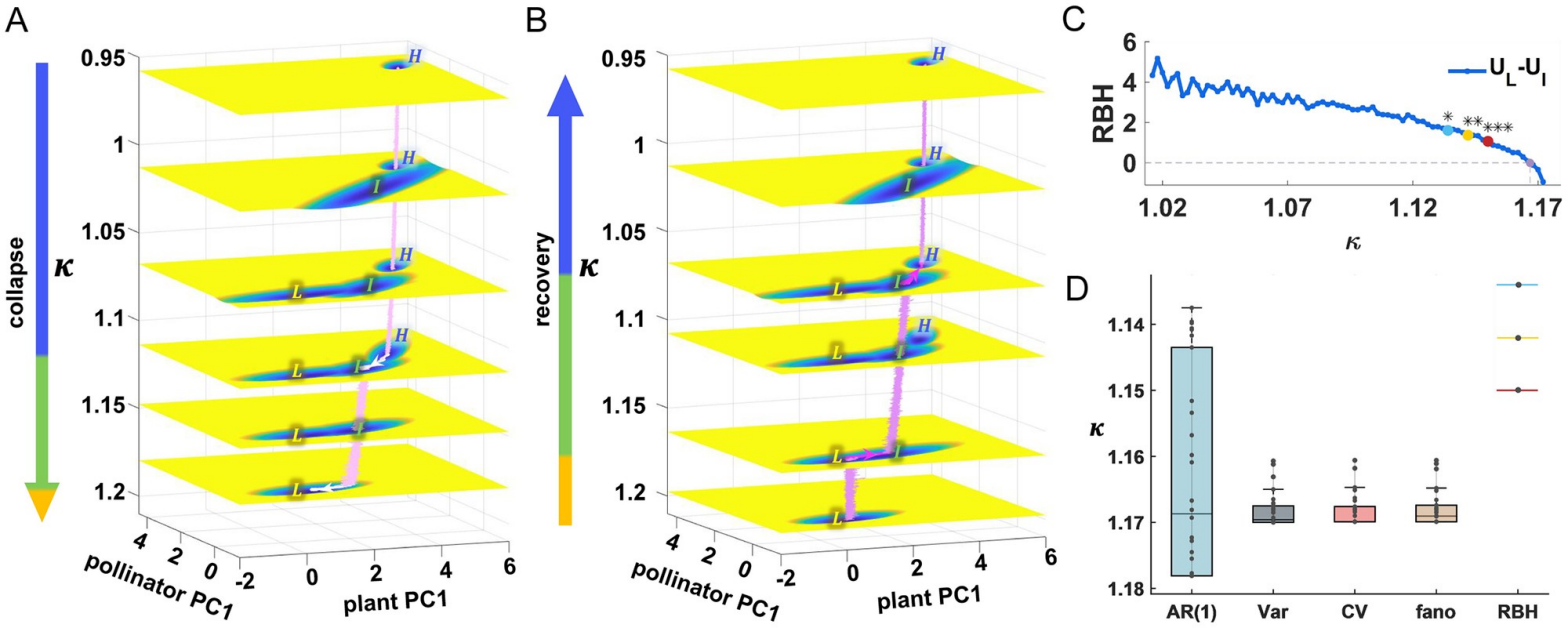
Barrier height serves as a new EWS to predict collapse. (A and B) The process of system collapse (A) and recovery (B) is simulated from the Langevin equation. We present two-dimensional landscape, in which the same coordinates are used to ensure comparability. The left arrow indicates the proportion of each state (H: blue, I: green, L: gold). The transitions between attractors are marked by thick arrows. (C) Calculated RBH between low and intermediate states with increasing *κ*. We regard the point as an EWS when the BDS statistic for the sequence up to this point is significant (blue: p<0.05, gold: p<0.01, red: p<0.001). (D) For complete collapse to the low state, the RBH can serve as the earliest warning signal compared with other metrics based on time series, such as AR(1), variance (Var), coefficient of variation (CV) and fano factor (fano). The color in RBH has the same meaning as (C), indicating the predicted critical *κ* value based on different p-values.

By simulating species abundance fluctuations as *κ* increases or decreases linearly with time, we found that the system undergoes transitions between attractors to achieve complete collapse (Fig 5A) or recovery (Fig 5B). The trajectories in abundance were calculated from multidimensional Langevin equations with parameter *κ* varying, and were then projected onto plant PC1 and pollinator PC1 obtained previously. We used $\kappa_{1}^{-}$ and $\kappa_{1}^{+}$ to denote the collapse and recovery thresholds between high and intermediate states respectively, as well as $\kappa_{2}^{-}$ and $\kappa_{2}^{+}$ to represent those between intermediate and low states analogously. Regarding the collapse process (Fig 5A), the system stayed in the high state despite subsequent appearance of tristability. It did not transit to the intermediate state until the high state was no longer a stable attractor ($\kappa_{1}^{-}\approx 1.11$). Confronted with the progressive decay, the system shifted toward the low state (the complete collapse occurred) when the intermediate state disappeared ($\kappa_{2}^{-}\approx 1.18$). We found that, for collapse, the system transits to a new stable state when its current state disappears as parameters change.

As for the recovery from the low state (Fig 5B), the system required better environmental conditions (smaller *κ*) while transiting to the intermediate state ($\kappa_{2}^{+}\approx 1.16$, $\kappa_{2}^{+}<\kappa_{2}^{-}$). For further restoration, it was stuck in the intermediate state longer (even if the high state emerged), and returned to the high state once more under better conditions ($\kappa_{1}^{+}\approx 1.07$, $\kappa_{1}^{+}<\kappa_{1}^{-}$). By comparing $\kappa_{1}^{+}$ with $\kappa_{1}^{-}$ and $\kappa_{2}^{+}$ with $\kappa_{2}^{-}$, we explored the emergence of hysteresis loops, which has been extensively mentioned in previous bistability studies [5, 10, 44, 45]. The system tends to remain in its current stable state. The transition to the new state occurs when the current state gradually becomes unstable and the system is driven by some noise, rather than transitioning as soon as the new state appears.

Unlike trajectory-based metrics, the landscape portrays the relative stability. As species abundance can exhibit strong nonlinear structures before tipping points [45, 46], we propose that RBH showing similar patterns can serve as an early-warning signal (EWS) of final collapse. To guarantee comparability, we selected the RBH between low and intermediate states (Fig 5C). With increasing *κ*, their corresponding RBHs can be seen as a sequence. We aimed to find the critical *κ*, when the sequence after adding a new point of RBH shows a nonlinear structure. We used the Brock-Dechert-Scheinkman (BDS) test on residuals after eliminating the effects of linear trends [47], and the null hypothesis is rejected at the critical point. Corresponding to three types of significance (*p* < 0.05, *p* < 0.01, *p* < 0.001), we marked three critical *κ*. In fact, after the disappearance of the high state, the RBH changes more drastically compared to that under smaller *κ*, which suggests that the earlier warning can be achieved.

Then we compared the results of RBH with traditional metrics (AR(1), variance, coefficient of variation and fano factor) derived from time series. Remarkably, RBH can act as the earliest warning signal, which is confirmed in multiple networks (Fig 5D and see Section E and F, Figs G, H and T in S1 Text for details). Since RBH is evaluated from a landscape picture, its corresponding critical *κ* is a definite value, not depending on individual species. Whereas traditional metrics rely on the individual trajectory, we took their median value for each species in comparison with RBH. The smaller the *κ* is, the earlier the warning is given. Moreover, the RBH is global and stable. It is not necessary to consider the selection of specific species, the choice of time windows and possible differences in each simulation. As for traditional metrics, we evaluated their ability to anticipate transitions based on multidimensional Langevin simulation trajectories after Gaussian filtering [45] (see Fig I in S1 Text for details). Our goal is to find the smallest possible *κ*, after which (i.e. for larger *κ*) the metrics continue to increase up to the tipping point of collapse. We also noticed that not all species can give accurate warnings in advance (Fig 5D and see Fig I in S1 Text for details), because most pollinator species (mainly specialists) suffered earlier extinctions before the intermediate state, as already mentioned in our multistability analysis (Fig 2C). Previously these species were confirmed to provide valuable information in predicting collapse [46, 48]. However, here we present a distinct perspective that those surviving in the intermediate state (mainly generalists) should be monitored, and their time series provide relatively accurate and early predictions (see Fig I in S1 Text for details).

Besides, when *κ* exhibits nonlinear increase, the predictive effectiveness of RBH remains significantly better than traditional indicators (Fig J and K in S1 Text). We also explored the situation when *κ* exhibits weakly autocorrelated fluctuations. We found that there was no statistically significant point in the RBH sequence to reject the BDS test (Fig L in S1 Text). This implies that there won’t be prediction of critical points when the transition does not occur, indicating that our EWS can to some extent address the issue of false positives.

### Global sensitivity analysis for parameters identifies the key role of pollinators surviving in the intermediate state

**Fig 6:**
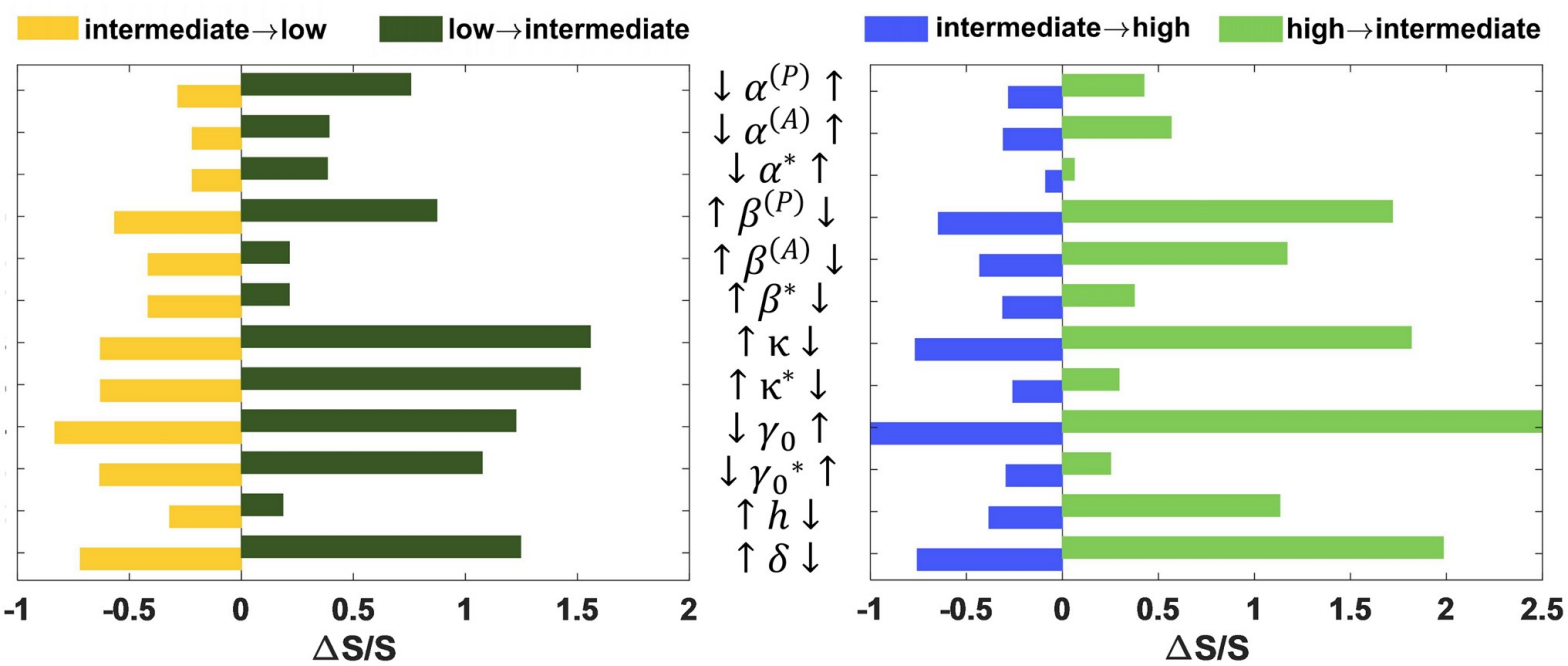
Global sensitivity analysis for parameters identifies the key factors. The parameters are perturbed 10% from the default value. The left panel corresponds to the transition between intermediate and low states, with parameter perturbation in the direction of system collapse, and the right panel represents the opposite perturbations. α*, β*, κ* and ${\gamma_{0}}^{*}$ denote perturbations of the only surviving pollinator in the intermediate state on the intrinsic growth rate, intraspecific competitions, average decay rate and per capita mutualistic strength respectively. Since rising 10% of *γ*_0_ makes the system exhibit high state only, the action is 0 for intermediate to high state transition, but infinity conversely. The corresponding relative changes are -1 and infinity (denoted as 2.5).

To see which parameters are critical to the state transitions in the plant-pollinator network, we perturbed each parameter by decreasing and increasing 10% from the default value under which the system exhibits tristability, and assessed the change of transition actions between states. What’s more, a strategy for ecological management is pinning the decay rate of the maximum degree pollinator at zero [32]. It inspires us to individually perturb the parameters related to this pollinator which survives alone in the intermediate state, denoted by $\alpha^{*},\beta^{*},\kappa^{*},{\gamma_{0}}^{*}$. It turns out that the intermediate state is retained after all perturbations except that rising *γ*_0_ generates only high state. Since perturbing the system towards collapse may cause the disappearance of high state, we quantified the relative change of transition actions between intermediate and low states when *α*^(*P*)^, *α*^(*A*)^, *γ*_0_ decreases or *β*^(*P*)^, *β*^(*A*)^, *κ*, *h*, *δ* increases for simplicity and consistency (Fig 6 left). Likewise, the same analysis is done between high and intermediate states when *α*^(*P*)^, *α*^(*A*)^, *γ*_0_ increases or *β*^(*P*)^, *β*^(*A*)^, *κ*, *h*, *δ* decreases (Fig 6 right).

On the whole, the reciprocal interaction parameters, *γ*_0_, *δ* and *κ*, have a strong influence on the evolutionary process. The system significantly shifts to the low state with greater *δ*, *κ* or less *γ*_0_ but to the high state with less *δ*, *κ* or greater *γ*_0_. An unanticipated finding is that separately perturbing the relevant parameters of the surviving pollinator ($\alpha^{*},\beta^{*},\kappa^{*},{\gamma_{0}}^{*}$) has similar effectiveness compared with perturbations on all species *α*^(*A*)^, *β*^(*A*)^, *κ*, *γ*_0_ during the transition between intermediate and low states. This clarifies the conclusions of [32] from a quantitative perspective, and again reminds us that promoting ecological management of the pollinators surviving in the intermediate state can effectively slow down the transition to low state so as to avoid sudden collapse without warning signals. However, as for high-intermediate transition, the role of this pollinator is relatively diminished. We realized that controlling a single pollinator is not sufficient to restore the system to high state, unless more favorable conditions are created. We should mention that pollinators surviving in the intermediate state are not always with larger degrees. Indeed it is more effective to protect these surviving pollinators than selected pollinators according to degree, discovered in other networks (see Section G, Fig M and N in S1 Text for details).

### Necessity of multidimensional models for generating intermediate state

There exists a general paradigm of dimensionality reduction for complex systems [5, 33]. The reduced model for plant-pollinator network is governed by two-dimensional (2D) dynamic differential equations, which has an excellent predictive efficacy of tipping points [8]. However, there is always a tradeoff between more detailed descriptions and more simplified analyzing approaches. Here, we argued for the necessity of multidimensional modelling in plant-pollinator networks, since we cannot discover the intermediate state in the 2D plant-pollinator equations by scanning the whole parameter space. We performed a linear stability analysis of the 2D reduced model in detail.

Firstly, we stated the reduced equations with two variables written in terms of *P*′ and *A*′ [8]:

$$\begin{array}{c} \begin{array}{cc} \frac{dP^{\prime }}{dt} & =\alpha P^{\prime }-\beta P{}^{\prime 2}+\frac{\langle \gamma_{P}\rangle A^{\prime }}{1+h\langle \gamma_{P}\rangle A^{\prime }}P^{\prime }+\mu ,\\ \frac{dA^{\prime }}{dt} & =\alpha A^{\prime }-\beta A{}^{\prime 2}-\kappa A^{\prime }+\frac{\langle \gamma_{A}\rangle P^{\prime }}{1+h\langle \gamma_{A}\rangle P^{\prime }}A^{\prime }+\mu . \end{array} \end{array}$$ (2)

We made the right-hand side of Eq 2 be zero to solve stationary points and obtained simplified forms with *μ* ≈ 0. We have (*P*_1_, *A*_1_) = (0, 0), $\left(P_{2},A_{2})=(0,\frac{\alpha -\kappa }{\beta }\right)$, $\left(P_{3},A_{3})=(\frac{\alpha }{\beta },0\right)$, and $(P_{*},A_{*})=(\frac{1}{\beta }(\alpha +\frac{\langle \gamma_{P}\rangle A^{\prime }}{1+h\langle \gamma_{P}\rangle A^{\prime }}),\frac{1}{\beta }(\alpha -\kappa +\frac{\langle \gamma_{A}\rangle P^{\prime }}{1+h\langle \gamma_{A}\rangle P^{\prime }})).$ The linear stability analysis is equivalent to investigating the Jacobian matrix, in fact, the system is stable if and only if all of Jacobian eigenvalues are with negative real parts. Because the intrinsic growth rate *α* is positive, (*P*_1_, *A*_1_) and (*P*_2_, *A*_2_) are always unstable. (*P*_3_, *A*_3_) is stable when $\kappa >\alpha +\frac{\langle \gamma_{A}\rangle \frac{\alpha }{\beta }}{1+h\langle \gamma_{A}\rangle \frac{\alpha }{\beta }}$, corresponding to our so-called low state (see Section H in S1 Text for details).

As for (P_*_, A_*_) (P_*_>0, A_*_>0), we first point out its explicit solution is two pairs of points: (*P*_4_, *A*_4_) and (*P*_5_, *A*_5_) (assume *A*_5_ ≥ *A*_4_). Specifically, by substituting *A*′ for *P*′, *A*′ satisfies the following quadratic equation:

$$\begin{array}{c} q_{1}A^{\prime 2}+q_{2}A^{\prime }+q_{3}=0, \end{array}$$ (3)

where

$$\begin{array}{c} \begin{array}{cc} q_{1}= & \beta^{2}h\langle \gamma_{P}\rangle +h^{2}\langle \gamma_{P}\rangle \langle \gamma_{A}\rangle \alpha \beta +h\beta \langle \gamma_{P}\rangle \langle \gamma_{A}\rangle ,\\ q_{2}= & \beta (h\langle \gamma_{A}\rangle \alpha +\beta )-\alpha h\langle \gamma_{P}\rangle \langle \gamma_{A}\rangle -\langle \gamma_{P}\rangle \langle \gamma_{A}\rangle +\\ & \left(\kappa -\alpha \right)(h\langle \gamma_{P}\rangle \beta +h^{2}\langle \gamma_{P}\rangle \langle \gamma_{A}\rangle \alpha +h\langle \gamma_{P}\rangle \langle \gamma_{A}\rangle ),\\ q_{3}= & \left(\kappa -\alpha \right)(h\langle \gamma_{A}\rangle \alpha +\beta )-\langle \gamma_{A}\rangle \alpha . \end{array} \end{array}$$

Here we focus on the change of *κ*, and analyses for other parameters are included in Section H, Fig O and Table B in S1 Text for details. As *κ* rises from zero, Eq 3’s axis of symmetry shifts to the left and the product of two roots *A*_4_*A*_5_ gradually increases to a positive value. *A*_5_ gradually becomes smaller but remains positive, and *A*_4_ grows larger from negative to positive, until they overlap at $\Delta =q_{2}^{2}-4q_{1}q_{3}=0$. If *κ* keeps soaring, Eq 3 will not exist a real solution. Therefore, the number of positive roots is from 1 to 2, then back to 1, and finally to 0. At Δ = 0, we can directly solve for the specific form of *A*′:

$$\begin{array}{c} A^{\prime }=\frac{\sqrt{\langle \gamma_{A}\rangle {\left\langle \gamma_{P}\right\rangle }^{3}}-\beta \langle \gamma_{P}\rangle -\alpha h\langle \gamma_{A}\rangle \langle \gamma_{P}\rangle }{h\langle \gamma_{A}\rangle {\left\langle \gamma_{P}\right\rangle }^{2}+\beta h{\left\langle \gamma_{P}\right\rangle }^{2}+\alpha h^{2}\langle \gamma_{A}\rangle {\left\langle \gamma_{P}\right\rangle }^{2}}. \end{array}$$ (4)

We discarded the larger solution of *κ* because the corresponding calculated *A*′ is negative.

Besides, (*P*_4_, *A*_4_) and (*P*_5_, *A*_5_) become stable under the condition that

$$\begin{array}{c} p_{1}A{}^{\prime 2}+p_{2}A^{\prime }+p_{3}>0, \end{array}$$ (5)

where

$$\begin{array}{c} \begin{array}{cc} & p_{1}={\left(\beta h\langle \gamma_{P}\rangle +h^{2}\langle \gamma_{P}\rangle \langle \gamma_{A}\rangle \alpha +h\langle \gamma_{P}\rangle \langle \gamma_{A}\rangle \right)}^{2},\\ & p_{2}=2(h\langle \gamma_{P}\rangle \beta +h^{2}\langle \gamma_{P}\rangle \langle \gamma_{A}\rangle \alpha +h\langle \gamma_{P}\rangle \langle \gamma_{A}\rangle )(\beta +h\langle \gamma_{A}\rangle \alpha ),\\ & p_{3}={\left(h\langle \gamma_{A}\rangle \alpha +\beta \right)}^{2}-\langle \gamma_{A}\rangle \langle \gamma_{P}\rangle . \end{array} \end{array}$$

Considering the negative axis of symmetry, we attained the positive solution of Eq 5 with ecological meaning:

$$\begin{array}{c} A^{\prime }>\frac{\sqrt{\langle \gamma_{A}\rangle {\left\langle \gamma_{P}\right\rangle }^{3}}-\beta \langle \gamma_{P}\rangle -\alpha h\langle \gamma_{A}\rangle \langle \gamma_{P}\rangle }{h\langle \gamma_{A}\rangle {\left\langle \gamma_{P}\right\rangle }^{2}+\beta h{\left\langle \gamma_{P}\right\rangle }^{2}+\alpha h^{2}\langle \gamma_{A}\rangle {\left\langle \gamma_{P}\right\rangle }^{2}}. \end{array}$$ (6)

Interestingly, the right-hand side of Eqs 4 and 6 is exactly the same. Owing to the impact of *κ* changes on the system, when Eq 3 has two positive roots, *A*_5_ is always stable but *A*_4_ is always unstable, whatever the value of *κ*. It is also possible to exist only one positive (not multiple) root *A*_5_, which must be stable. So Eq 3 corresponds with a unique stable state (*P*_5_, *A*_5_) all the time, namely the high state.

In conclusion, at most two stable states, high state (*P*_5_, *A*_5_) and low state (*P*_3_, *A*_3_) mentioned above, are discovered in the 2D model based on linear stability analysis. It is somewhat surprising that the intermediate state is not detected here, which makes us lose the opportunity for deeper analysis of possible mechanisms. Therefore, we propose that a multidimensional model is necessary for exploring the dynamics in plant-pollinator network, where intermediate state may play critical roles in state transitions.

## Discussion

Transient phenomena in ecology have long aroused wide concern, but the precise underlying ecological mechanisms have yet to be fully explored [5, 8, 49]. The intermediate state, uncovered by simulating species dynamics in multidimensional systems, reveals a new perspective concerning the evolution of plant-pollinator networks. For a representative network that exhibits tristability, we leverage the landscape framework to characterize its relative stability and transient behaviour [12, 50]. Our major findings include: (*i*) During the transition from high to low state, the system tends to go through the intermediate state, discovered both in calculated transition paths within the tristable range and in simulated trajectories under environmental degradation. (*ii*) The BH, calculated from the landscape, has the best performance in predicting final collapse compared with traditional metrics based on time series. (*iii*) The species that survive in the intermediate state play a central role in keeping the ecosystem away from collapse, but a limited role in full recovery.

**Fig 7:**
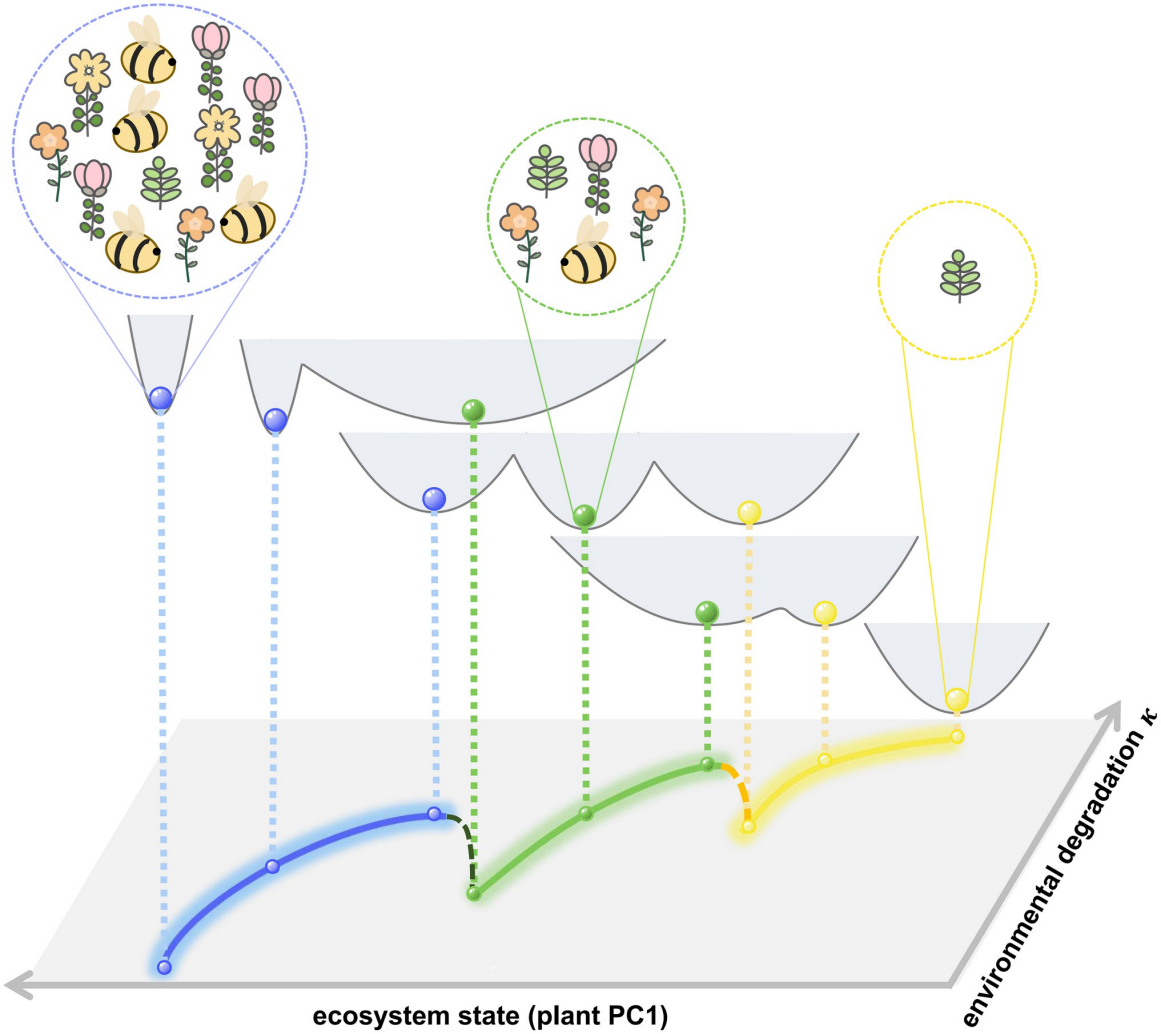
Changes in ecosystem state from ongoing environmental degradation illustrated by potential energy landscape. The global decay in pollinators caused by the damage results in landscape topography changes, and further leads to transitions between stable states. The ecosystem state is represented by the value of plant PC1, and the true potential energy is obtained from actual simulations. The phase diagram versus *κ* is also shown.

We propose that the system proceeds through the intermediate state before collapse. At such a transition state, more than half pollinators suffer extinctions (defined as abundance below 1e-3) and the remaining species reluctantly maintain the ecological functionality. Under fixed parameters within the tristable domain, we discovered the indispensable role of intermediate states based on both projected and multidimensional transition paths between attractors (Fig 2B and 2C). The paths also indicate that collapse first occurs in pollinators, which can help to guide ecological management. As a non-equilibrium system with non-gradient forces, the plant-pollinator network is susceptible to external perturbations [51, 52]. The environmental degradation not only straightforwardly makes some species (mainly pollinators) vulnerable to extinction [20, 21, 53], but also triggers declines in pollinator abundance for a given network. The latter is modelled by a linear term in the dynamic equations that pollinators satisfy (Eq 1). For the case of variable parameters, we mainly focused on *κ*, as well as *γ*_0_. Following previous work [8, 10, 32, 35], we assume that the *κ* is the same for all pollinators. Considering *κ* to be different for each pollinator would result in more complex analysis, which warrants further investigation. We found that the system goes through the intermediate state either from the landscape change (Fig 3) or from the trajectory simulation (Fig 5A). The re-emergence of the hysteresis loops reflects the irreversibility of transitions once again. The restoration from the low state requires more favorable conditions (smaller *κ*) by comparison to tipping points of collapse, both recovering to intermediate state and to high state (Fig 7).

Resilience is a central concept in ecology [5], and currently we use quantitative tools to portray it. The classic ball and cup explanation intuitively illustrates the difficulty of transitions between two alternative attractors [44, 54]. The reduced-dimensional approach for landscape we proposed is essentially a more detailed and adequate description of the system state, where we selected two interpretable and robust coordinates. Our generalized approach is particularly applicable to multistable systems, and here the coordinate value can roughly reflect population changes in plant and pollinator abundance. We conclude that either degrading the environment or decreasing the reciprocal strength allows the system to collapse along different pathways (Fig 3). It depends on specific parameters, for example, larger *κ* inevitably generates a low stable state. The reduced landscapes globally portray the change of each attractor, which is quantified by the BH. BH can quantitatively characterize the stability and resilience of the ecosystem. With changing *κ*, BH shows consistent tendency with transition action and MFPT that both are derived from multidimensional models (Fig 4).

We aim to find an EWS for final collapse, allowing for the anticipation of ecosystem collapse, identification of potential biodiversity loss risks, and the implementation of preventive measures to mitigate the collapse. The well-known critical slowing-down phenomena appear prior to the phase transition, manifested by increased autocorrelation and variance in species abundance [45, 55]. Consistent with previous studies [46, 48], we also noticed specialist pollinators become extinct sooner, so they are believed to anticipate critical transitions earlier. However, due to massive extinction of pollinators in the intermediate state, they probably give false warnings of complete collapse (see Fig I in S1 Text for details). The BH, as a global measure of landscape, exhibits a nonlinear structure in the pre-collapse period (Fig 5C). Remarkably, with particular regard to complete collapse, it has the best early warning effectiveness than time-series metrics (Fig 5D). In addition, we propose that the pollinators that survive in the intermediate state, should be monitored as keystone species, which means they enable earlier and more accurate warnings. From a global sensitivity analysis, we pointed out pinning the relevant parameters of these surviving pollinators can effectively mitigate the collapse, but it does little to full recovery (Fig 6). Besides, we explained why a multidimensional model is necessary. The collective behaviors in biological communities can hardly be described by individual variable, since it inhibits understanding of potential biological mechanisms [56]. Based on the theory of linear stability, we underlined that the heterogeneity in species interactions should be fully considered, which is necessary for the discovery of intermediate states.

Overall, the intermediate state suggests that, apart from sudden ecosystem collapse after crossing a tipping point, there remains another possibility that the collapse is a gradual process. It may pass through a ‘bridge’, which has been found and experimentally confirmed in microbial communities [56] and gene regulation networks [57, 58]. Regarding the reasons for the emergence of intermediate states here, inspired by the gene regulatory network studies where positive feedbacks promote the emergence of multistability [59], we argue that plant-pollinator system exhibits the intermediate state due to the presence of more positive feedback loops in the multidimensional model.

Remarkably, the BH quantified from the landscape, serves as a prospective EWS to anticipate final collapse. We need to stress that, currently the barrier height is obtained from the quantified landscape, which requires a dynamical model. However practically, what we are provided with is the raw experimental or observational data, which requires us to reconstruct the landscape according to the data without the need for prior model assumptions. In principle, the landscape can be obtained from the observational time series data through the collection of the joint statistics or using other data-driven approaches [60, 61]. By integrating data collected from multiple plots with frequent sampling [53, 62, 63], we can obtain available samples to reconstruct the landscape. Just as applying time windows to calculate traditional indicators, we can estimate the landscape using observational data of each species within the time window. By sliding the time window along long-term trajectories, we can separately reconstruct the landscape at multiple time points, and then calculate their BH to achieve early warning.

Except the typical trisable state case, we also studied different network scenarios for considering the heterogeneity of the reciprocal interaction in different networks (see Section I in S1 Text for details). Our claims regarding BH as EWS and indispensable role of species in intermediate states also hold for other tristable networks (see Fig G, M, P-U and Tables C, D in S1 Text for details) and tetrastable networks (see Fig H, N and Table E in S1 Text for details). The landscape and transition path approach provides a general framework to study stochastic transition dynamics in ecosystems, and our results help to develop better management strategies to achieve environmental sustainability.

## Methods

### Model of plant-pollinator mutualistic networks

We assume that an ecological network consists of *N* = *N*_*P*_ + *N*_*A*_ species in total, including *N*_*P*_ plants and *N*_*A*_ pollinators. All parameters in Eq 1 except *γ* are set to be node-independent (except for the perturbations in global sensitivity analysis). The intrinsic growth rate $\alpha_{i}^{\left(P\right)}\left(\alpha_{j}^{\left(A\right)}\right)$ excludes the effects of mutualism and competitions. We regard the intraspecific competitions $\beta_{ii}^{\left(P\right)}\left(\beta_{jj}^{\left(A\right)}\right)$ are substantially stronger than the interspecific competitions $\beta_{ik}^{\left(P\right)}\left(\beta_{jm}^{\left(A\right)}\right)$ [9, 35]. The environmental degradation causes global pollinator decline expressed through average decay rate *κ*. The mutualistic interaction is modelled through the nonlinear functional response as a particular case of the Hill function with the power exponent of 1. If both plants and pollinators exhibit high abundance, the saturation effect will be manifested. It is denoted by half-saturation constant *h* and also interpreted as handling time [64], which corresponds to Holling second type functional response [65]. The reciprocal strength $\gamma_{jn}^{\left(P\right)}$ is similarly defined as $\gamma_{jn}^{\left(P\right)}=\gamma_{0}\varepsilon_{jn}/{\left[g_{j}^{\left(A\right)}\right]}^{\delta }$. Here *ε*_*jn*_ indicates whether a link exists between the *j*^*th*^ pollinator and the *n*^*th*^ plant, $g_{j}^{\left(A\right)}$ is the degree of the *j*^*th*^ pollinator. Meanwhile, the tradeoff between reciprocal intensity and number of interactions should be taken into account by *δ*(0 ≤ *δ* ≤ 1). For *δ* = 0, all links share the same average strength without considering network topology. For *δ* = 1, the strength is strongly dependent upon the connection number, when it is weakened in the species with more links. Here we choose *δ* = 0.5 which follows the same value as previous work [8, 10, 32]. The immigration rate *μ* is close to zero and neglected in our network dynamics.

### Landscape quantified through Truncated Moment Equation (TME)

The evolution trajectory of ecosystem can be described by the Langevin equation, in which the drift term does not depend explicitly on time *t* and the random term *ζ* is included to depict the fluctuation:

$$\begin{array}{c} \frac{dx(t)}{dt}=f\left(x\right)+\zeta \left(t\right), \end{array}$$

where $x\left(t\right)={\left[P_{1},\ldots ,P_{N_{P}},A_{1},\ldots ,A_{N_{A}}\right]}^{T}$, $f\left(x\right)={\left[F_{1},\ldots ,F_{N_{P}},G_{1},\ldots ,G_{N_{A}}\right]}^{T}$ corresponding to the right-hand side of Eq 1, and ***ζ*** = [*ζ*_1_(*t*), …, *ζ*_*N*_(*t*)]^*T*^. We preset 〈***ζ***(*t*)〉 = **0** since nonzero mean can be absorbed into *f*(***x***), as well as the element of covariance matrix 〈*ζ*_*i*_(*t*), *ζ*_*j*_(*t*′)〉 = 2*dδ*_*ij*_*δ* (*t* − *t*′) in which no correlation is revealed between different times or components. *d* represents the diffusion coefficient (noise intensity), *δ*_*ij*_ is an indicator function and *δ* denotes the Dirac Delta function. Furthermore, as ***x***(*t*) has continuous sample paths, Fokker-Planck equation (FPE) could also reflect the dynamic change of probability as an equivalent form. The probability density function *p*(***x***, *t*∣***x***_0_, *t*_0_) satisfies

$$\begin{array}{c} \begin{array}{c} \partial_{t}p(x,t|x_{0},t_{0})=-\sum_{i}\;\partial_{i}[f_{i}\left(x\right)p(x,t|x_{0},t_{0})]+d\;\sum_{i,j}\partial_{i}\;\partial_{j}[p(x,t|x_{0},t_{0})]. \end{array} \end{array}$$ (7)

We choose the initial condition given by *p*(***x***, *t*_0_ ∣ ***x***_0_, *t*_0_) = *δ*(***x*** − ***x***_0_) and the boundary condition as a reflecting barrier, i.e. $\vec{n}\cdot J\left(x,t\right)=0$ for ***x*** ∈ boundary, where $\vec{n}$ is the normal vector of the boundary, and the component of probability current ***J*** is defined as *J*_*i*_(***x***, *t*) = *f*_*i*_(***x***)*p*(***x***, *t* ∣ ***x***_0_, *t*_0_) − *d* ∑_*j*_ ∂_*j*_*p*(***x***, *t* ∣ ***x***_0_, *t*_0_). It guarantees zero net flow of probability across the boundary.

On account of the nonlinear term in drift force, it is unrealistic to derive the analytic solution of Eq 7. Based on the *Ω* expansion theory [66, 67], we have developed a truncated moment equation approach to approximately solve FPE, where we ignore the impact of the third and higher order moments on probability function under the condition of *d* ≪ 1. Hence the actual evolution of system is approximated by Gaussian distribution along a deterministic trajectory, whose mean $\bar{x}\left(t\right)$ and covariance matrix Σ(*t*) satisfy the following ordinary differential equations [13, 15, 68]:

$$\begin{array}{cc} \dot{\bar{x}}\left(t\right)= & f\left(\bar{x}\right),\\ \dot{\Sigma }\left(t\right)= & \Sigma \left(t\right)A^{T}\left(t\right)+A\left(t\right)\Sigma \left(t\right)+2d\cdot I, \end{array}$$

in which *A*(*t*) is the Jacobian of ***f***(***x***) at $x=\bar{x}\left(t\right)$, whose element is calculated by $A_{ij}\left(t\right)={\frac{\partial }{\partial x_{j}}f_{i}\left(x\right)|}_{x=\bar{x}\left(t\right)}$. Thus we obtain the approximate solution of Eq 7:

$$\begin{array}{c} p\left(x\left(t\right),t\right)=\frac{\text{exp}(-\frac{1}{2}{\left(x\left(t\right)-\bar{x}\left(t\right)\right)}^{T}\Sigma^{-1}\left(t\right)\left(x\left(t\right)-\bar{x}\left(t\right)\right))}{{\left(2\pi \right)}^{\frac{N}{2}}{\left|\Sigma (t)\right|}^{\frac{1}{2}}}. \end{array}$$

Then each attractor’s stationary density *p*_*ss*_(***x***) is acquired when convergence is reached at sufficiently large *t*. Furthermore, for a system with multistability, we will assume that the global stationary density function is a sum of weighted Gaussian mixtures, i.e. $p_{ss}\left(x\right)=\sum_{j=1}^{M}\phi^{j}p_{ss}^{j}\left(x\right),$ where $p_{ss}^{j}\left(x\right)$ is the stationary solution of ***x***^*j*^, its weight *ϕ*^*j*^ is evaluated by the frequency after sampling multiple random initial conditions, and *M* is the number of stable states. As $\sum_{j=1}^{M}\phi^{j}=1$, *p*_*ss*_(***x***) is also a probability density function. Ultimately, the potential energy landscape is quantified through *U* = −ln *p*_*ss*_(***x***) [13, 15].

### Generalized dimension reduction approach for landscape

First, we denote plants by $P={\left[P_{1},\ldots ,P_{N_{P}}\right]}^{T}$ and pollinators by $A={\left[A_{1},\ldots ,A_{N_{A}}\right]}^{T}$. After the TME method, we obtain the multidimensional stationary probability density function $p_{ss}\left(P,A\right)=\sum_{j=1}^{M}\phi^{j}p_{ss}^{j}\left(P,A\right),$ where $p_{ss}^{j}\left(P,A\right)$ follows a multivariate Gaussian distribution *N*(*μ*^*j*^(*P*, *A*), Σ^*j*^(*P*, *A*)).

Through integrating *A* or *P*, we get the marginal probability density function of *P* or *A*. To avoid redundancy, we write *X* to express *P* or *A*. So $p_{ss}\left(X\right)=\sum_{j=1}^{M}\phi^{j}p_{ss}^{j}\left(X\right)$, $\mu \left(X\right)=\sum_{j=1}^{M}\phi^{j}\mu^{j}\left(X\right)$ and $\Sigma \left(X\right)=\sum_{j=1}^{M}\phi^{j}\left(\Sigma^{j}\left(X\right)+\mu^{j}\left(X\right)\mu^{j}{\left(X\right)}^{T}\right)-\mu \left(X\right)\mu {\left(X\right)}^{T}$ (see Section C in S1 Text for details). Then we use the singular value decomposition of Σ(*P*) and Σ(*A*) independently, with the aim of finding their own first PC. It is also referred to as the eigenvector corresponding to the largest eigenvalue and written as *w*_1_(*P*) and *w*_1_(*A*). Afterwards, we project the original high-dimensional system onto the two new directions, among which the x-axis is defined as *z*_1_ = *w*_1_(*P*)^*T*^*P* and the y-axis is defined as *z*_2_ = *w*_1_(*A*)^*T*^*A*. Both of them obey Gaussian distributions which are calculated from *z*_1_ ∼ *N*(*w*_1_(*P*)^*T*^*μ*(*P*), *w*_1_(*P*)^*T*^Σ(*P*)*w*_1_(*P*)) and *z*_2_ ∼ *N*(*w*_1_(*A*)^*T*^*μ*(*A*), *w*_1_(*A*)^*T*^Σ(*A*)*w*_1_(*A*)) respectively. In other words, if we denote $W={\left[\begin{array}{cc} w_{1}\left(P\right) & 0_{N_{P}\times 1}\\ 0_{N_{A}\times 1} & w_{1}\left(A\right) \end{array}\right]}_{N\times 2}$ and *z* = [*z*_1_, *z*_2_], we can attain the joint probabilistic density function after dimension reduction, which is a two-dimensional Gaussian distribution *z* ∼ *N*(*W*^*T*^*μ*(*P*, *A*), *W*^*T*^Σ(*P*, *A*)*W*). The reduced energy landscape can be computed from *U* = −ln (*p*_*z*_) accordingly.

This framework is particularly powerful for systems in which variables from two classes separately satisfy two different forms of equations, while equations of the same type are fulfilled for variables within class. An advantage of this approach is that new coordinates has their concrete practical meaning, since x-axis is a linear combination of plants and y-axis is a linear combination of pollinators. Instead of generating the second PC which contributes little to the total variance, our approach is more reliable and valid, and weights in coordinates remain relatively robust in our searching parameters. Simultaneously, we further confirm that the first PC is significantly superior to other PCs in Σ(*P*), Σ(*A*) and Σ(*P*, *A*) (see Fig D in S1 Text for details).

### The transition action calculation

The transition action between ***x***^*i*^ and ***x***^*j*^ quantitatively characterizes the transition feasibility from one attractor to another. With specified final time *T*, we denote a transition path by $x^{ij}\left(t\right)={\left[x_{1}^{ij}\left(t\right),\ldots ,x_{N}^{ij}\left(t\right)\right]}^{T}$ for *t* ∈ [0, *T*], which follows the boundary conditions ***x***^*ij*^(0) = ***x***^*i*^ and ***x***^*ij*^(*T*) = ***x***^*j*^. Then, for each path, its transition action is defined as

$$\begin{array}{c} S^{ij}(x^{ij})=\frac{1}{2}\int_{0}^{T}{{∥{\left(\frac{dx_{1}^{ij}\left(t\right)}{dt},\ldots ,\frac{dx_{N}^{ij}\left(t\right)}{dt}\right)}^{T}-f(x^{ij}\left(t\right))∥}_{2}}^{2}dt. \end{array}$$

According to the Wentzell-Freidlin theory [69], the action is proportion to the negative logarithm of the probability of ***x***(*t*). Therefore, all we need to do is to minimize the transition action over all possible paths in the original high-dimensional system. The adaptive minimum action method [70, 71] is used to optimize this problem.

## Supporting information

S1 Text Supporting Information for “Landscape quantifies the intermediate state and transition dynamics in ecological networks”. Description of our simulation details, analysis procedures, additional figures and tables.
(PDF)
