# Supplementary material for: Landscape quantifies the intermediate state and transition dynamics in ecological networks
Source: PLoS Comput Biol. 2024 Jan 5;20(1):e1011766. doi: 10.1371/journal.pcbi.1011766 (PMC10796024; doi:10.1371/journal.pcbi.1011766)
Supplement: S1 Text — Description of our simulation details, analysis procedures, additional figures and tables. (PDF) [file pcbi.1011766.s001.pdf]

S1 Text: Supporting Information for
"Landscape quantifies the intermediate state and transition dynamics in ecological networks"

Jinchao Lv, Jin Wang, Chunhe Li

This PDF file includes:
Section A to I
References
Figs A to U
Tables A to E

A. Noise-induced stable state transitions

Here we demonstrate that, with fixed parameters, noise can also induce transitions between stable states. We keep the parameter κ fixed and add noise with $d = 0.003$. Taking $\kappa = 1.12$, by initiating the system from the low state (Fig Aa) and the high state (Fig Ab), we observe that the system transits to the intermediate state, which is induced by the noise. Similarly, taking $\kappa = 1.02$, by initiating the system from the low state (Fig Ac) and the intermediate state (Fig Ad), we observe that the system transits to the high state. As for this recovery process, our calculated transition path indicates that plants recover earlier than pollinators (Fig 2C), and we also observe this phenomenon in the stochastic trajectories. Presented by plant 3 and pollinator 3, with initial points in the low state without external force, the plants recover earlier than the pollinators (Fig Ae). However, if we add the external intervention, such as keeping the pollinator 2 at high abundance, the plants will also recover earlier than pollinators (Fig Af).

We also consider the scenario where $\zeta(t)$ represents colored noise (red noise and blue noise). The red noise and blue noise are generated using an autoregressive (AR) method [1]: $\varepsilon_t = \lambda\varepsilon_{t-1} + \omega_t$, where λ is the autocorrelation coefficient (red noise: $\lambda = 0.95$, blue noise: $\lambda = -0.95$) and ω_t is a standard normal random component (mean = 0, variance = 1). We referred to the internal noise forms provided by Meng [2] and constructed the potential landscape of the system through Langevin equation simulations (Fig C). The landscape is quantified through statistical ensemble distributions of trajectories that are simulated from different initial points over a sufficiently long period of time. We found that in the case of red noise, transitions between stable states are more likely to occur, in other words, the red noise promotes the system's escape from the current attractor. This is because the transition trend in a certain direction is intensified at the next time point. While in the case of blue noise, transitions are less likely to occur due to the negative correlations between adjacent time points.

B. Details of settings for ordinary differential equations (ODEs)

To obtain Fig 1B and construct the landscape, we need to solve for the stable states of the ODEs (Eq.1). We utilized the Runge-Kutta method with variable step size to

simulate forward using MATLAB's ode45 solver, which is a high-order algorithm for solving ODEs. The initial conditions involve randomly and uniformly distributing points within a feasible high-dimensional range (all species abundances are non-negative and bounded). We considered the system to have reached stability when the Euclidean distance between species abundance vectors in adjacent steps is less than 1e-7. As for the landscape, the weights for each attractor are obtained based on the proportion of points falling on each after uniform sampling.

C. Generalized dimension reduction approach for landscape

Here we show in detail the mathematical derivation of our dimension reduction approach and its advantages. Our approach is an improvement over previous work that proposed a general framework to perform a dimension reduction for landscape [3]. We have modelled that the multidimensional stationary probability density function is

$$p_{ss}(P, A) = \sum_{j=1}^{M} \phi^j p_{ss}^j(P, A), \tag{1}$$

where the symbol meanings are detailed in the main text. We initially applied this approach and selected the first two eigenvector directions for projections, to maximize the variance of population probability distribution after projection. However, we found that it is not very suitable for this bipartite system.

First, the biological meanings of two coordinate variables are vague. The first principal component (PC1) and the second principal component (PC2) both are composed of plants and pollinators with equally important and indispensable weights (Figs Da-b and Ec-d). They further result that the reduced landscape can not adequately present the information we need. For example, the transition paths between stable states can provide the order information of species extinction and recovery, and we expect that the projected paths in the reduced landscape can roughly retain the information, at least about plant and pollinator population. But we cannot get this by applying this method. We also noted that PC2 is perhaps redundant, where its value of the intermediate state may be larger (Fig Dc) or smaller (Fig Dd) than that of the low state. This is related to the negative weights of the components that make up PC2 (Figs Da-b). Moreover, when we perturbed the value of parameter, we found that two coordinate variables (PC1 and PC2) exhibit a drastic change (Figs Da-b), which directly leads to a significant difference between their corresponding landscapes (Figs Dc-d). Here we changed the value of κ and fixed other parameters under default values. We showed the results of weights as well as their landscapes at $\kappa = 1.09$ (Figs Da and Dc) and $\kappa = 1.1$ (Figs Db and Dd). We concluded that the previous approach may undergo significant changes in variable weights and landscapes after small perturbations of parameters, i.e., it is not considered robust enough.

To address the above shortcomings and to take into account the plant pollinator-networks which are composed of two sets of nodes, we developed a novel dimension reduction approach for landscape. Inspired by hierarchical principal component analysis [4], the variables in this bipartite Network Can be naturally divided into two segments (plants and pollinators). We took the derivation of the plant population (denoted by $P = [P_1, \ldots, P_{S_P}]^T$) as an example, and the conclusions of pollinator population (denoted by $A = [A_1, \ldots, A_{S_A}]^T$) can be simply reached by replacing P with A. We first obtained its stationary probability density function

through integrating A in Eq.S1:

$$\begin{aligned}
p_{ss}(P) &= \int_0^{+\infty} \cdots \int_0^{+\infty} p_{ss}\left(P_1,\ldots,P_{N_P},A_1,\ldots,A_{N_A}\right) dA_1 \cdots dA_{N_A} \\
&= \int_0^{+\infty} \cdots \int_0^{+\infty} \sum_{j=1}^{M} \phi^j p_{ss}^j\left(P_1,\ldots,P_{N_P},A_1,\ldots,A_{N_A}\right) dA_1 \cdots dA_{N_A} \\
&= \sum_{j=1}^{M} \phi^j \int_0^{+\infty} \cdots \int_0^{+\infty} p_{ss}^j\left(P_1,\ldots,P_{N_P},A_1,\ldots,A_{N_A}\right) dA_1 \cdots dA_{N_A} \\
&= \sum_{j=1}^{M} \phi^j p_{ss}^j\left(P_1,\ldots,P_{N_P}\right) \\
&= \sum_{j=1}^{M} \phi^j p_{ss}^j(P).
\end{aligned}$$

Then the expectation of component P_i is derived as follows:

$$\begin{aligned}
\mu\left(P_i\right) &= \int_0^{+\infty} \cdots \int_0^{+\infty} P_i p_{ss}\left(P\right) dP_1 \cdots dP_{N_P} \\
&= \int_0^{+\infty} \cdots \int_0^{+\infty} P_i \sum_{j=1}^{M} \phi^j p_{ss}^j(P) dP_1 \cdots dP_{N_P} \\
&= \sum_{j=1}^{M} \phi^j \int_0^{+\infty} \cdots \int_0^{+\infty} P_i p_{ss}^j(P) dP_1 \cdots dP_{N_P} \\
&= \sum_{j=1}^{M} \phi^j \mu^j\left(P_i\right).
\end{aligned}$$

So the expectation of vector P is

$$\mu(P) = \sum_{j=1}^{M} \phi^j \mu^j(P),$$

where $\mu^j(P)$ is the jth attractor's Gaussian mean of the plant segment.

We are more concerned with the covariance matrix between variables, whose element σ_{pq} (p^{th} row, q^{th} column) can be calculated by

$$\begin{aligned}
\operatorname{Cov}\left(P_p,P_q\right) &= E\left[\left(P_p - EP_p\right)\left(P_q - EP_q\right)\right] \\
&= E\left(P_p P_q\right) - EP_p \cdot EP_q \\
&= \int_0^{+\infty} \cdots \int_0^{+\infty} P_p P_q p_{ss}(P) dP_1 \cdots dP_{N_P} - \sum_{j=1}^{M} \phi^j \mu^j\left(P_p\right) \cdot \sum_{j=1}^{M} \phi^j \mu^j\left(P_q\right) \\
&= \int_0^{+\infty} \cdots \int_0^{+\infty} P_p P_q \sum_{j=1}^{M} \phi^j p_{ss}^j(P) dP_1 \cdots dP_{N_P} - \sum_{j=1}^{M} \phi^j \mu^j\left(P_p\right) \cdot \sum_{j=1}^{M} \phi^j \mu^j\left(P_q\right) \\
&= \sum_{j=1}^{M} \phi^j \int_0^{+\infty} \cdots \int_0^{+\infty} P_p P_q p_{ss}^j(P) dP_1 \cdots dP_{N_P} - \sum_{j=1}^{M} \phi^j \mu^j\left(P_p\right) \cdot \sum_{j=1}^{M} \phi^j \mu^j\left(P_q\right) \\
&= \sum_{j=1}^{M} \phi^j \left(\Sigma^j\left(P_p,P_q\right) + \mu^j\left(P_p\right)\mu^j\left(P_q\right)\right) - \sum_{j=1}^{M} \phi^j \mu^j\left(P_p\right) \cdot \sum_{j=1}^{M} \phi^j \mu^j\left(P_q\right),
\end{aligned}$$

where $\Sigma^j(P_p, P_q)$ is the (p,q) element of the jth attractor's Gaussian covariance matrix of the plant segment ($N_P \times N_P$ submatrix in the upper left corner). The last equation comes from the definition of covariance:

$$\begin{aligned}\Sigma^j(P_p, P_q) &= E^j(P_pP_q) - \mu^j(P_p)\,\mu^j(P_q) \\ &= \int_0^{+\infty}\cdots\int_0^{+\infty} P_pP_qp_{ss}^j(P)dP_1\cdots dP_{N_P} - \mu^j(P_p)\,\mu^j(P_q).\end{aligned}$$

Therefore, the covariance matrix is obtained from:

$$\Sigma(P) = \sum_{j=1}^{M}\phi^j\left(\Sigma^j(P) + \mu^j(P)\otimes\mu^j(P)^T\right) - \mu(P)\otimes\mu(P)^T,$$

where $\otimes$ is the Kronecker product. We can directly substitute A into B obtain the equations satisfied by pollinators. Then we separately selected the PC1 of plant covariance matrix and pollinator covariance matrix as new coordinates, denoted by plant PC1 and pollinator PC1 respectively. They are computed using singular value decomposition (SVD), and essentially the eigenvector corresponding to the largest eigenvalue of each two matrices.

Although current deviation form is similar to that in the previous approach [3], we will point out that they are fundamentally different. Our approach is more applicable to this bipartite system. First, for high-dimensional systems where both N_P and N_A are very large, it is more computable to solve the eigenvector corresponding to the largest eigenvalue of two lower dimensional matrices ($N_P \times N_P$ and $N_A \times N_A$), compared to solving the eigenvectors corresponding to the first two eigenvalues of a high-dimensional matrix ($(N_P + N_A) \times (N_P + N_A)$). Next, our novel coordinates are composed of linear combinations of plants and pollinators respectively, which ensures interpretability (Figs De, Df, Ea and Eb). We can roughly infer the overall plant behavior from plant PC1 and pollinators as well. Meanwhile, the almost non-negative weights indicate that, in the reduced landscape, large values of plant PC1 and pollinator PC1 correspond to the high state, while small values correspond to the low state. The same perturbation demonstrates the stability of our approach, where neither the weights in two coordinate variables nor the reduced landscapes change significantly (Figs De-h).

Furthermore, for wider range of parameters ($\kappa \sim U[1.01, 1.12]$), we depicted changes in weights of each species that make up plant PC1 (Fig Ea), pollinator PC1 (Fig Eb), PC1 (Fig Ec) and PC2 (Fig Ed). During the variation period, we also showed the distribution of variance contribution rate of plant PC1 in $\Sigma(P)$, pollinator PC1 in $\Sigma(A)$, as well as PC1 and PC2 in $\Sigma(P, A)$ (Fig Ee). Our two coordinates (plant PC1 and pollinator PC1) are much more robust, and their combination contains the information of PC1 (Figs Ea-c). Meanwhile, they are more interpretable, which can describe overall evolution of plant and pollinator population but the individual PC1 can not. It is intuitive to choose two coordinates to visualize the landscape, which can provide as much detail as possible. We also noticed that PC2 varies dramatically, and thus not easy to choose the reference coordinates (Fig Ed). Indeed, by demonstrating the absolute dominance of plant PC1 in $\Sigma(P)$, pollinator PC1 in $\Sigma(A)$, PC1 in $\Sigma(P, A)$, combined with the little contribution rate of PC2 in $\Sigma(P, A)$ (Fig Ee), we reemphasized that PC2 does not provide valid information and also showed the superiority of our approach.

D. Calculation of transition action and MFPT and their consistency with barrier height (BH)

We first select an appropriate L to calculate the transition action. Smaller L results in a less smooth path, for example, no optimization performed when L is set to 2, which

means that two stable points are directly connected with a straight line in high-dimensional space. On the other hand, larger L can lead to computational difficulties and potentially unstable calculations. Based on computational considerations, we opt for an appropriate value of L and keep it fixed for subsequent analysis. We demonstrate the impact of varying L on the transition action and computational time (Fig B), and we choose $L = 20$ to balance the principle of least action and computation time.

Considering that the saddle point calculated in the high-dimensional system may not correspond well to that calculated in the reduced landscape, we chose RBH as a substitution indicator for BH in the main text. Moreover, the point with the local minimum potential energy in the high-dimensional system is nearly always projected onto the attractor (also with local minimum potential energy) in the reduced landscape. This correspondence implies that RBH can adequately describe the evolution of the system. Here we confirmed the strong relationship between ΔS and RBH (Fig Fa). We can observe their positive correlation, in fact, they show very similar trends with κ varying (Figs 4A-F). The anomaly occurs at smaller κ, during which the weight of high state is much greater than that of the intermediate and low states. This complete dominance of the high state will make the calculation of RBH biased.

To calculate the MFPT in our multidimensional system, it is challenging to solve $f(\boldsymbol{x}) \cdot \nabla\tau + d\nabla\tau \cdot I \cdot \nabla\tau = -1$. A feasible alternative solution is based on the trajectory simulation from equivalent high-dimensional Langevin equations $\frac{d\boldsymbol{x}(t)}{dt} = \boldsymbol{f}(\boldsymbol{x}(t)) + \boldsymbol{\zeta}(t)$. We treated a supra-sphere as a proxy of an attractor, where its center is quantified from the stable-state mean in our TME method, and radius is the hyperparameter r we specified. For each experiment, we randomly picked an initial point in the supra-sphere and then simulated a trajectory through the Euler-Maruyama method. The timer of first passage time (FPT) is started when it initially leaves $\boldsymbol{x}^i$, and stopped when it first arrives in $\boldsymbol{x}^j$. We repeated 100 experiments and then averaged their FPT to approximate the MFPT. We simulated for long enough time, trying to capture the transition process. We recorded MFPT of three feasible transitions under different parameter κ ($\kappa = 1.02 \sim 1.07$: intermediate to high, $\kappa = 1.02 \sim 1.06$: low to high, $\kappa = 1.08 \sim 1.16$: low to intermediate, all at 0.01 interval). Under Euclidean distance, we tuned the hyperparameter r, and showed the change of MFPT with varying κ (Fig Fb). We chose $r = 0.5$ in our main text, but similar tendency indicates that its selection does not affect our general conclusions. As κ rises, the time required to transit to the state with higher abundance is longer. We clarified the approximate linear relationships between RBH and the logarithm of MFPT (log(MFPT)), as well as transition action and MFPT, choosing $r = 0.3$ (Fig Fc) and $r = 0.4$ (Fig Fd). Similar linear correlations were found, again validating the robustness to the selection of hyperparameter r and the consistency of these three metrics.

E. The plant-pollinator network topologies can generate complex multistability

In our main text, we chose a representative network that can achieve tristability by varying parameters. Its nodes and links are detailedly shown in Table A. Here we are only concerned that whether there is communication between plants and pollinators, instead of the number of specific visits observed.

Actually, Fig 1B has shown that far more than this sub-Network can generate an intermediate state, during which various proportions of pollinators remain alive. However, different intermediate states share the following characteristics: (a) The abundance of plants is generally at a moderate level. (b) More than half of the

pollinators go extinct and the surviving species are typically generalists that have more connections with plants. Then we represent two additional scenarios for sub-networks, still obtained by removing pollinators from the orginal network (Hickling, Norfolk, UK, M_PL_006) [5].

Network A (17 plants and 25 pollinators) is derived from removal of 60% pollinators (Table C), which also generates tristable behaviour (Fig Ga). A proportion of plants (P11, P13, P14, P15 and P17) are never pollinated by pollinators. The difference here is that more pollinators (40%) survive in the intermediate state. We also notice the abundance of some plants in the intermediate state is close to that in the high state, since higher pollinators richness and abundance promote plant diversity. This fact suggests that it may not be appropriate to project the landscape onto certain species. Not surprisingly, different patterns may emerge, including slightly higher abundance of some plants in the low state and the extinction of a few pollinators in the high state. These are due to the effect of intraspecific competition.

Network C (17 plants and 13 pollinators) is obtained from removal of 80% pollinators (Table E), taken as an example of the emergence of two intermediate states (intermediate I & II, I_1&I_2) (Fig Ha). Both of them have characteristics mentioned previously, and the one closer to the high state (intermediate II, I_2) is featured by more abundant and more diverse pollinator communities (Fig Ha). In fact, only one species (L6) differs significantly, which survives in I_2 but goes extinct in I_1. Here six plants (P11, P12, P13, P14, P15 and P16) that do not have connections to pollinators (Table E).

Our numerical simulations reveal that those species surviving in the intermediate state do not individually die out with parameter varying, they go extinct together when the intermediate state disappears and transits to the low state. Thus the intermediate state is some kind of parameter-independent, intrinsic property. Besides, we propose that the pollinator species surviving in the intermediate state are not always those with larger degree. For Network A, ten pollinators (I1, I4, I5, I8, I9, I11, I12, I14, I16 and I17 in Table C) survive in the intermeidate state, among which I9's degree is 1. The degrees of I2, I3 and I6 are all 2, but they all go extinct in the intermeidate state. For Network C, three pollinators (L1, L2 and L6 in Table E) survive in the intermeidate state, their degree are 8, 4 and 3 repectively. L9's degree is 4, but it goes extinct in the intermeidate state.

F. RBH has better predictive efficiency than traditional metrics based on stochastic time series

We still focused on the variation of parameter κ, and first considered that it increases or decreases linearly with time. We were interested in the region where the phase transition occurs and simulated the trajectory of abundance change for each species during $\kappa = 0.96 \sim 1.2$. We chose $dt = 0.01$, steps=2.4×10^5, $d = 5 \times 10^{-4}$, $\kappa = 0.96 + 10^{-6} \times i^{th}$step for collapse and $\kappa = 1.2 - 10^{-6} \times i^{th}$step for recovery. Based on the parameter-varying Langevin equation, we simulated the abundance fluctuation of thirty species. Then it was projected onto the previously obtained coordinates (plant PC1 and pollinator PC1) and the occurrence of two phase transitions can be clearly seen (Figs 5A and B).

The early warning is concerned with the collapse process, also known as the transition to the low state. After we applied Gaussian filtering to each species trajectory (Figs Ia-b), we obtained the residuals' autocorrelation at lag 1 (AR(1)) and variance (Var), which are calculated in a time window (5% datapoints) to represent the value of a single point. The coefficient of variance (CV) and fano factor (fano) are both

quantified from original species abundance, since we considered that it leads to a smaller denominator after filtration. These indicators show an upward trend before the system undergoes the phase change [6, 7]. Our procedures are shown and illustrated in terms of AR(1). For each species corresponding trajectory, the goal is to find the smallest κ_0 that satisfies $\text{AR}(1)(\kappa_0) < \text{AR}(1)(\kappa)$ for all $\kappa \in (\kappa_0, \kappa_*)$, where κ_* represents the tipping point of collapse. It is equivalent to consider $\text{slope}_{\text{AR}(1)}(\kappa) > 0$ for all $\kappa \in (\kappa_0, \kappa_*)$ [7]. Based on least-squares linear regression over 20% datapoints of AR(1), we estimated their slopes and found the minimum κ_0 according to the above criterion. We calculated the minimum κ_0 corresponding to all species. Then the procedures are repeated for other metrics (Var, CV, fano) (Fig 5D).

Based on these metrics, the early warning is possible for some species, especially for pollinators surviving in the intermediate state (pollinator 2). For three species (plant 3, plant 15 and pollinator 2), we showed the variation of AR(1) (Fig Ic) and variance (Fig Ie) with varying κ. For each species and metric, we also marked the location of the minimum κ_0 (red points in Figs Ic and Ie). However, we found that some species (like plant 6, pollinator 3 and pollinator 8) cannot provide accurate early-warning signals (EWSs) (Figs Id and If), i.e., no so-called κ_0 exists (grey points in Figs Id and If). A possible explanation is that most pollinators become extinct before the intermediate state. Their degrees are generally lower, but that's not always the case, like Network A and Network C. Their extinction does not mean the collapse of the system, and they do not exhibit obvious characteristics in terms of system collapse. The traditional perspective is that species with lower degrees are feasible to anticipate critical transitions because they go extinct earlier [7]. We did find this pattern, but here the real breakdown is marked by the extinction of pollinators surviving in the intermediate state (generally with larger degrees), and their monitoring is more effective.

Derived from our reduced landscape, the RBH can be seen as a sequence with varying κ. We desired to find the critical point when the whole sequence up to this point exhibits nonlinearity. The Brock-Dechert-Scheinkman (BDS) test is applied to detect it [8]. We eliminated linear trends using the first difference, and then performed the test on its residuals. They first need to accept the hypothesis that they are white noise, by the Ljung-Box Q-test (LBQ test). The collapse transition induced by strong nonlinear responses is expected to reject the BDS's null hypothesis that residuals are independent and identically distributed [6, 8]. After comparisons with traditional metrics obtained before, the RBH's critical point is much earlier (smaller κ). We also performed above analysis for Network A (Figs Gb-e) and Network C (Figs Hb-e), the hysteresis loops always appear (Figs Gb-c and Hb-c). In general, RBH serves as the earliest warning signal of collapse, except for choosing $p < 0.001$ in Network C (red line in Fig He). By the way, through collapse and recovery trajectory in Network A, we found that the collapse process goes through the intermediate state (Fig Gb), while recovery does not (Fig Gc).

Besides, when κ exhibits nonlinear changes, the predictive effectiveness of RBH remains significantly better than traditional indicators (Figs J and K). We also explored the situation when κ exhibits weak autocorrelated fluctuations ($\kappa(\mathbf{t+1}) = \omega(\mathbf{t+1}) +$ 1. 1, where $\omega(\mathbf{t+1}) = 0.3 \times \omega(\mathbf{t}) + \boldsymbol{\xi}(\mathbf{t})$ and $\omega(\mathbf{0}) = \mathbf{0}, \boldsymbol{\xi}(\mathbf{t}) \sim \boldsymbol{N}(\mathbf{0}, \mathbf{1/40})$), the BDS test does not reject the null hypothesis, indicating that the EWS can to some extent mitigate the issue of false positives (Fig L).

G. Global sensitivity analysis identifies the indispensable role of species surviving in the intermediate state

Here we further illustrate the importance of intermediate state species for mitigating collapse, by analyzing the relative change of transition action based on global sensitivity analysis. As we did in our main text, we selected a set of parameters in the domain of multistability, and made suitable perturbations, trying to ensure the presence of intermediate states. Then we calculated the relative change in the transition action, to quantitatively portray the evolution of the system. Despite the network discussed in the main text, the only surviving pollinator in the intermediate state do have the largest degree. But it is not always the case. For example, for Network A, ten pollinators survive in the intermediate state, in which the degree of I9 (1) is less than the degrees of extinct pollinators I2, I3 and I6 (2). For Network C, three pollinators survive in the intermediate state, in which the degree of L6 (3) is less than the degree of extinct pollinator L9 (4).

We next showed that it is more efficient to pin parameters related to those surviving in the intermediate state, rather than choosing species by degree. After ranking the degrees of pollinators, to ensure comparability, we perturbed parameters of top ten pollinators (with top ten degrees) in Network A and top three pollinators (with top three degrees) in Network C. For Network A, there exists a tie. We randomly chose I2, I3, I5, I6, I11 in terms of degree=2, and the remaining species chosen by degrees are I1 (7), I4 (5), I8 (3), I12 (3) and I16 (4). We labeled the species degrees in parentheses. The most generalist species is I1 (7), we also separately perturbed parameters associated with it to assess the impact of this individual species. We showed again the surviving species: I1 (7), I4 (5), I5 (2), I8 (3), I9 (1), I11 (2), I12 (3), I14 (2), I16 (4) and I17 (2). For Network C, the species chosen by degrees are L1 (8), L2 (4) and L9 (4), the most generalist species is L1 (8), and the surviving species are L1 (8), L2 (4) and L6 (3).

We perturbed parameters of these three types (chosen by degree, generalist and surviving in the intermediate state), and marked the superscript 'degree', 'generalist' and 'our' respectively. For comparison we again did the case of perturbing all parameters, where no superscripts are labelled. We applied suitable (small) perturbations (Network A: 1%, Network C: 0.5%), trying to avoid the phase change. We calculated the relative changes of transition actions of intermediate to low transition and intermediate to high transition (Network A: Fig M, Network C: Fig N).

For Network A, we found that, in the case of increasing 1% of γ_0 and decreasing 1% of κ, the intermediate state disappears and is fully converted to the high state (Fig M). The transition action from intermediate to high state is 0 and its relative change is -1; the action from intermediate to low state is infinity and its relative change is infinity. Remarkably, we again emphasized that, for intermediate to low transition (gold bar in Fig M), perturbing species surviving in the intermediate state ('our' in Fig M) has almost the same effect as perturbing all species (no superscripts in Fig M). Good ecological management of these species can essentially mitigate the collapse. It is also more effective than perturbing the same number of species based on degree ('degree' in Fig M), as well as singly perturbing the most generalist species ('generalist' in Fig M). Surviving species are associated with the complex network topology, and they cannot be directly inferred from the magnitude of the degree. The most generalist pollinator plays a certain but relatively limited role. However, for full recovery (intermediate to high transition, blue bar in Fig M), the effectiveness of perturbation on above three types becomes smaller, especially for the individual generalist pollinator. As we discussed in our main text, recovery to the high state is a joint result of interspecific collaboration, and the role played by a few pollinators is restricted.

For Network C, although four stable states can appear, we noticed that the intermediate II appears in a small region during our perturbation experiments. Also considering the consistency of studies in Network A, we focused on the transition actions from intermediate I to low state and intermediate I to high state, and tentatively referred to intermediate I as the intermediate state. We found that, in the case of increasing and decreasing 0.5% of γ_0, the intermediate state disappears, which confirms the influence of the reciprocity parameter on the system once again (Fig N). We noted that the intermediate state is fully converted to the low state at decreasing 0.5% of γ_0, while to the high state at increasing 0.5% of γ_0. For the former case, the transition action from intermediate to low state is 0 and its relative change is -1; the action from intermediate to high state is infinity and its relative change is infinity. For the latter case, the relative change is infinity for the intermediate to low transition, and is -1 for the intermediate to high transition. The important role of species surviving in the intermediate state for collapse mitigation is discovered again (Fig N). The conclusion that controlling a few pollinators has a limited role in the transition to the high state is also consistent (Fig N).

H. Linear stability analysis demonstrates that intermediate states only appear in multidimensional frameworks

The dimension reduction approach is much challengeable since we always have to strike a better balance between simplified modelling and detailed descriptions. Prior research has noted how to predict tipping points in plant-pollinator mutualistic networks through reduced two-dimensional (2D) equations [9]. Nevertheless, the main weakness of the paradigm is that we cannot identify stable intermediate states via all suitable parameters. To the best of our knowledge, this is the first time to analyze the linear stability of the 2D reduced models in detail.

We again showed the 2D equations with respect to P' and A' [9]:

$$\begin{aligned} \frac{dP'}{dt} &= \alpha P' - \beta P'^2 + \frac{\langle \gamma_P \rangle A'}{1 + h \langle \gamma_P \rangle A'} P' + \mu, \\ \frac{dA'}{dt} &= \alpha A' - \beta A'^2 - \kappa A' + \frac{\langle \gamma_A \rangle P'}{1 + h \langle \gamma_A \rangle P'} A' + \mu. \end{aligned} \tag{2}$$

where definitions for most parameters $(\alpha, \beta, h, \mu, \kappa)$ is the same as in multidimensional model. Additionally, $\langle \gamma_P \rangle = \gamma_0 \frac{\sum_{i=1}^{S_P} k_i^{2-\delta}}{\sum_{i=1}^{S_P} k_i}, \langle \gamma_A \rangle = \gamma_0 \frac{\sum_{j=1}^{S_A} d_j^{2-\delta}}{\sum_{j=1}^{S_A} d_j}$, in which k_i and d_j are nodal degrees of the ith plant and j^{th} pollinator respectively. To solve the stationary points, the right-hand sides of Eq.S2 are set equal to zero. Then we obtained:

$$\begin{aligned} P' &= -\frac{1}{2\beta} \left(-\alpha - \frac{\langle \gamma_P \rangle A'}{1 + h \langle \gamma_P \rangle A'} \pm \sqrt{\left(\alpha + \frac{\langle \gamma_P \rangle A'}{1 + h \langle \gamma_P \rangle A'} \right)^2 + 4\beta\mu} \right), \\ A' &= -\frac{1}{2\beta} \left(-\alpha + \kappa - \frac{\langle \gamma_A \rangle P'}{1 + h \langle \gamma_A \rangle P'} \pm \sqrt{\left(\alpha - \kappa + \frac{\langle \gamma_A \rangle P'}{1 + h \langle \gamma_A \rangle P'} \right)^2 + 4\beta\mu} \right), \end{aligned}$$

where the sign $\pm$ can be either positive or negative. Thus we had four possible combinations $(++), (+-), (-+), (--)$. We presented the simplified forms with $\mu \approx 0$:

$(P_1, A_1) = (0, 0)$, $(P_2, A_2) = \left(0, \frac{\alpha-\kappa}{\beta}\right)$, $(P_3, A_3) = \left(\frac{\alpha}{\beta}, 0\right)$ and $(P_*, A_*) = \left(\frac{1}{\beta}\left(\alpha + \frac{\langle\gamma_P\rangle A'}{1+h\langle\gamma_P\rangle A'}\right), \frac{1}{\beta}\left(\alpha - \kappa + \frac{\langle\gamma_A\rangle P'}{1+h\langle\gamma_A\rangle P'}\right)\right)$. However, this does not mean four pairs of points. (P_*, A_*) $(P_* > 0, A_* > 0)$ actually corresponds to two pairs of points, as A_* is the solution of a quadratic equation (Eq. **3** in the main text).

Therefore, we can analyze the linear stability of above four pairs by their corresponding Jacobian matrices. Since the intrinsic growth rate remains positive ($\alpha > 0$), at least one eigenvalue of $J_{(P_1,A_1)} = \begin{bmatrix} \alpha & 0 \\ 0 & \alpha-\kappa \end{bmatrix}$ has a positive real part. By Lyapunov's first method, (P_1, A_1) is always unstable. As for (P_2, A_2), the eigenvalues of $J_{(P_2,A_2)} = \begin{bmatrix} \alpha + \frac{\langle\gamma_P\rangle\frac{\alpha-\kappa}{\beta}}{1+h\langle\gamma_P\rangle\frac{\alpha-\kappa}{\beta}} & 0 \\ \langle\gamma_A\rangle\frac{\alpha-\kappa}{\beta} & \kappa-\alpha \end{bmatrix}$ are $\alpha + \frac{\langle\gamma_P\rangle\frac{\alpha-\kappa}{\beta}}{1+h\langle\gamma_P\rangle\frac{\alpha-\kappa}{\beta}}$ and $\kappa - \alpha$. They cannot be both negative, which results in the unstable (P_2, A_2). However, (P_3, A_3) can become stable on condition that $\kappa > \frac{\langle\gamma_A\rangle\frac{\alpha}{\beta}}{1+h\langle\gamma_A\rangle\frac{\alpha}{\beta}} + \alpha$, by calculation of $J_{(P_3,A_3)} = \begin{bmatrix} -\alpha & \langle\gamma_P\rangle\frac{\alpha}{\beta} \\ 0 & \frac{\langle\gamma_A\rangle\frac{\alpha}{\beta}}{1+h\langle\gamma_A\rangle\frac{\alpha}{\beta}} + \alpha - \kappa \end{bmatrix}$. Indeed, it corresponds to the low state we considered in our multidimensional model. Here the reduced model is in good agreement with the original multidimensional model (Fig 1D). All pollinators went extinct ($A_3 = 0$) and all plants showed similar patterns of low abundance which can be approximated by $P_3 = \frac{\alpha}{\beta}$. This also implies that increasing κ will inevitably make the low state stable, both for the reduced and multidimensional models.

Although we have analyzed (P_*, A_*) in the main text, next we show more details. First, its Jacobian matrix is $J(P_*, A_*) = \begin{bmatrix} -\alpha - \frac{\langle\gamma_P\rangle A'}{1+h\langle\gamma_P\rangle A'} & \frac{\langle\gamma_P\rangle\left(\alpha+\alpha h\langle\gamma_P\rangle A'+\langle\gamma_P\rangle A'\right)}{\beta(1+h\langle\gamma_P\rangle A')^3} \\ \frac{\langle\gamma_A\rangle A'}{\left(1+\frac{1}{\beta}h\langle\gamma_A\rangle\left(\alpha+\frac{\langle\gamma_P\rangle A'}{1+h\langle\gamma_P\rangle A'}\right)\right)^2} & -A'\beta \end{bmatrix}$. Since $-\alpha - \frac{\langle r_P\rangle A'}{1+h\langle r_P\rangle A'}$ is always negative, (P_*, A_*) is stable if and only if the Jacobian determinant is positive. Then we derived the condition (Eq. **5**). By simulation of the effects of increasing κ from zero (in the main text), we have proved that (P_5, A_5) corresponding to larger root of Eq. **3** is always stable, and A_5 has a lower bound (Eq. **6**):

$$A_5 > \frac{\sqrt{\langle\gamma_A\rangle\langle\gamma_P\rangle^3} - \beta\langle\gamma_P\rangle - \alpha h\langle\gamma_A\rangle\langle\gamma_P\rangle}{h\langle\gamma_A\rangle\langle\gamma_P\rangle^2 + \beta h\langle\gamma_P\rangle^2 + \alpha h^2\langle\gamma_A\rangle\langle\gamma_P\rangle^2}.$$

Here our perspective is that the critical point (lower bound) of the high state (P_5, A_5) is an intrinsic property, which depends on the internal parameters and network, instead of external environmental parameter κ. We solved the critical value of κ which corresponds to the lower bound of A_5:

$$\begin{aligned}\kappa_1 =& \Big(h\langle\gamma_P\rangle^2(\beta + \langle\gamma_A\rangle + \alpha h\langle\gamma_A\rangle)\Big)^{-1}\Big(\beta^2\langle\gamma_P\rangle + \langle\gamma_A\rangle\langle\gamma_P\rangle^2 - 2\beta\sqrt{\langle\gamma_A\rangle\langle\gamma_P\rangle^3} \\ &+\alpha^2h^2\langle\gamma_A\rangle\langle\gamma_P\rangle^2 + \alpha\beta h\langle\gamma_P\rangle^2 + 2\alpha h\langle\gamma_A\rangle\langle\gamma_P\rangle^2 + \alpha\beta h\langle\gamma_A\rangle\langle\gamma_P\rangle\Big).\end{aligned}$$

It is derived from $\Delta = q_2^2 - 4q_1q_3 = 0$, in which the larger root

$$\begin{aligned}\kappa_2 =& \Big(h\langle\gamma_P\rangle^2(\beta + \langle\gamma_A\rangle + \alpha h\langle\gamma_A\rangle)\Big)^{-1}\Big(\beta^2\langle\gamma_P\rangle + \langle\gamma_A\rangle\langle\gamma_P\rangle^2 + 2\beta\sqrt{\langle\gamma_A\rangle\langle\gamma_P\rangle^3} + \\ &\alpha^2h^2\langle\gamma_A\rangle\langle\gamma_P\rangle^2 + \alpha\beta h\langle\gamma_P\rangle^2 + 2\alpha h\langle\gamma_A\rangle\langle\gamma_P\rangle^2 + \alpha\beta h\langle\gamma_A\rangle\langle\gamma_P\rangle\Big)\end{aligned}$$

is discarded because its corresponding abundance

$$A_5' = -\frac{\sqrt{\langle\gamma_A\rangle\langle\gamma_P\rangle^3} + \beta\langle\gamma_P\rangle + \alpha h\langle\gamma_A\rangle\langle\gamma_P\rangle}{h\langle\gamma_A\rangle\langle\gamma_P\rangle^2 + \beta h\langle\gamma_P\rangle^2 + \alpha h^2\langle\gamma_A\rangle\langle\gamma_P\rangle^2}$$

is negative, without any ecological meaning. It can also explain the appearance of bistability and hysteresis loops. When κ approaches its critical point κ_1, pollinator spcies abundance is close to its lower bound. Continuous increase in κ leads to the disappearance of the high state, and before that, the low state has met the conditions for reaching stability. It should be pointed out that the exact abundance in the high stable state is influenced by κ. We currently denote the critical point of κ by internal parameters, with the aim of better explanation and simpler forms. Other representations may result in lengthy calculations, but do not affect the results.

To facilitate analysis, we investigated the effect of controlling individual parameter. We have already discussed the case of κ in the main text. As for other parameters, we first took full advantage of κ_1's form, which can be regarded as a function with respect to α, β, h and γ_0. The emphasis here is the sign of the partial derivative has not changed in the domain we cared about. Here $\frac{\partial}{\partial\alpha}\kappa_1$ and $\frac{\partial}{\partial\gamma_0}\kappa_1$ are positive, but $\frac{\partial}{\partial\beta}\kappa_1$ and $\frac{\partial}{\partial h}\kappa_1$ are negative. We added the weak assumption ($\beta < \langle\gamma_A\rangle < \langle\gamma_P\rangle$) for convenience in the proof, which are always satisfied in our cases. The conclusions can be reached through arithmetic–geometric mean inequality, and neglecting small terms $O(h^2)$ since the default value of h is 0.2. We calculated that

$$\begin{aligned}
\frac{\partial}{\partial\alpha}\kappa_1 =& \Big(\langle\gamma_P\rangle^2(\beta+\langle\gamma_A\rangle+\alpha h\langle\gamma_A\rangle)^2\Big)^{-1}\Big(\langle\gamma_P\rangle^2\langle\gamma_A\rangle^2 + 3\beta\langle\gamma_P\rangle^2\langle\gamma_A\rangle + \beta\langle\gamma_P\rangle\langle\gamma_A\rangle^2 + \\
& 2\beta\langle\gamma_A\rangle\sqrt{\langle\gamma_A\rangle\langle\gamma_P\rangle^3} + \alpha^2h^2\langle\gamma_P\rangle^2\langle\gamma_A\rangle^2 + 2\alpha h\langle\gamma_P\rangle^2\langle\gamma_A\rangle^2 + 2\alpha\beta h\langle\gamma_P\rangle^2\langle\gamma_A\rangle + \\
& \beta^2\langle\gamma_P\rangle^2\Big) > 0, \\
\frac{\partial}{\partial\beta}\kappa_1 =& \Big(h\langle\gamma_P\rangle^2(\beta+\langle\gamma_A\rangle+\alpha h\langle\gamma_A\rangle)^2\Big)^{-1}\big[\langle\gamma_P\rangle(\beta^2-\langle\gamma_A\rangle\langle\gamma_P\rangle) + 2\langle\gamma_A\rangle\langle\gamma_P\rangle\times \\
& (\beta-\sqrt{\langle\gamma_A\rangle\langle\gamma_P\rangle})(\alpha h+1) + \alpha h\langle\gamma_A\rangle\langle\gamma_P\rangle(\langle\gamma_A\rangle-\langle\gamma_P\rangle) + h^2\alpha^2\langle\gamma_A\rangle^2\langle\gamma_P\rangle\Big] < 0, \\
\frac{\partial}{\partial h}\kappa_1 =& -\Big(h^2\langle\gamma_P\rangle^2(\beta+\langle\gamma_A\rangle+\alpha h\langle\gamma_A\rangle)^2\Big)^{-1}\big[(\langle\gamma_P\rangle\langle\gamma_A\rangle + \beta\langle\gamma_P\rangle + 2\alpha h\langle\gamma_P\rangle\langle\gamma_A\rangle)\times \\
& \Big(\langle\gamma_P\rangle\langle\gamma_A\rangle + \beta^2 - 2\beta\sqrt{\langle\gamma_P\rangle\langle\gamma_A\rangle}\Big) + h^2\Big(\alpha^2\beta\langle\gamma_P\rangle\langle\gamma_A\rangle^2 + \alpha^2\langle\gamma_P\rangle^2\langle\gamma_A\rangle^2\Big)\Big] < 0, \\
\frac{\partial}{\partial\gamma_0}\kappa_1 =& \beta\Big(\langle\gamma_P\rangle^2h\gamma_0\sqrt{\langle\gamma_P\rangle\langle\gamma_A\rangle}(\beta+\langle\gamma_A\rangle+\alpha h\langle\gamma_A\rangle)^2\Big)^{-1}\Big[\langle\gamma_A\rangle\langle\gamma_P\rangle\alpha h\sqrt{\langle\gamma_P\rangle\langle\gamma_A\rangle}\times \\
& \Big(\langle\gamma_P\rangle - \langle\gamma_A\rangle + 2\Big(\sqrt{\langle\gamma_A\rangle)\gamma_P\rangle} - \beta\Big)\Big) + (\langle\gamma_P\rangle\langle\gamma_A\rangle - \beta^2)\sqrt{\langle\gamma_A\rangle\langle\gamma_P\rangle^3} - \\
& h^2\langle\gamma_A\rangle^2\alpha^2\sqrt{\langle\gamma_A\rangle\langle\gamma_P\rangle^3} + 2\langle\gamma_P\rangle\langle\gamma_A\rangle\sqrt{\langle\gamma_P\rangle\langle\gamma_A\rangle}\Big(\sqrt{\langle\gamma_P\rangle\langle\gamma_A\rangle} - \beta\Big)\Big] > 0.
\end{aligned}$$

This implies to us that changing parameters in one direction causes monotonic effects in κ_1. Worse conditions (smaller α, γ_0 or larger β, h) make the κ_1 smaller, and the high stable state disappers when κ_1 is smaller than current κ.

The monotonic impacts of increasing parameters on other variables (A_5 lower bound, the axis of symmetry, $A_4 * A_5$ and A_5) can be found similarly (Table B), and we omit the lengthy details. Here o indicates that increasing value of the variable has no effect on the first-row variable, + represents increased parameters have positive effects on either increasing value or moving right, − represents negative effects and / denotes the effect is not verified. An unexpected finding is the opposite effect of α and γ_0 on the lower bound, where it decreases when α increases, but increases when γ_0 increases.

These continuous and monotonic changes ensure that A_5 is the only stable point over a large parameter range. Numerical simulations also show the expected and consistent results (Fig O). We considered cases of varying individual parameter and fixed other parameters that do not affect the shape of the function. Varying multiple parameters is also a matter of changing individual parameters in sequence. Overall, worse conditions cause A_5 to approach its lower bound, also the dividing point between stability and instability, but A_4 never goes beyond it. So far, we have reported a basic understanding of the reduced 2D system. It has at most two stable states, (P_3, A_3) and (P_5, A_5), over an appropriate and widespread range of parameters.

I. A new network to replicate our findings

We reproduce our conclusions in a new network in the Web-of-Life database [10] (Fig Pa). Similarly, by removing a subset of pollinators, we identified a subnetwork that exhibits an intermediate state (Network B: Fig Pb, Table D), enabling us to describe the abundance of each species in stable states (Fig Pc). Next, we plotted the energy landscape (Fig Qa) in reduced coordinates (Fig Qb) and calculated the transition paths between stable states (Fig Qc), once again revealing a tendency to transition from the high state to the low state through the intermediate state.

We have also demonstrated the landscape change with parameter variation (Fig R), the consistency between RBH and transition action (Fig S), and RBH as an EWS (Fig T). Sensitivity analysis once again highlights the role of pollinators surviving in the intermediate state in preventing system collapse (Fig U). Here, the pollinators surviving in the intermediate state happen to be the two largest-degree pollinators.

References

1. Ripa J, Lundberg P. Noise colour and the risk of population extinctions. Proceedings of the Royal Society of London Series B: Biological Sciences. 1996;263(1377): 1751–1753.

2. Meng Y, Jiang J, Grebogi C, Lai YC. Noise-enabled species recovery in the aftermath of a tipping point. Physical Review E. 2020; 101(1): 012206

3. Kang X, Li C. A Dimension Reduction Approach for Energy Landscape: Identifying Intermediate States in Metabolism-EMT Network. Advanced Science. 2021;8(10):2003133.

4. Wold S, Kettaneh N, Tjessem K. Hierarchical multiblock PLS and PC models for easier model interpretation and as an alternative to variable selection. Journal of chemometrics. 1996;10(5-6):463–482.

5. Dicks L, Corbet S, Pywell R. Compartmentalization in plant-insect flower visitor webs. Journal of Animal Ecology. 2002;71(1):32–43.

6. Dakos V, Carpenter SR, Brock WA, Ellison AM, Guttal V, Ives AR, et al. Methods for detecting early warnings of critical transitions in time series illustrated using simulated ecological data. PloS one. 2012;7(7):e41010.

7. Aparicio A, Velasco-Hernández JX, Moog CH, Liu YY, Angulo MT. Structure-based identification of sensor species for anticipating critical transitions. Proceedings of the National Academy of Sciences. 2021;118(51):e2104732118.

8. Broock WA, Scheinkman JA, Dechert WD, LeBaron B. A test for independence based on the correlation dimension. Econometric reviews. 1996;15(3):197–235.

9. Jiang J, Huang ZG, Seager TP, Lin W, Grebogi C, Hastings A, et al. Predicting tipping points in mutualistic networks through dimension reduction. Proceedings of the National Academy of Sciences. 2018;115(4):E639–E647.

10. Motten AF. Pollination ecology of the spring wildflower community of a temperate deciduous forest. Ecological Monographs. 1986;56(1):21–42.

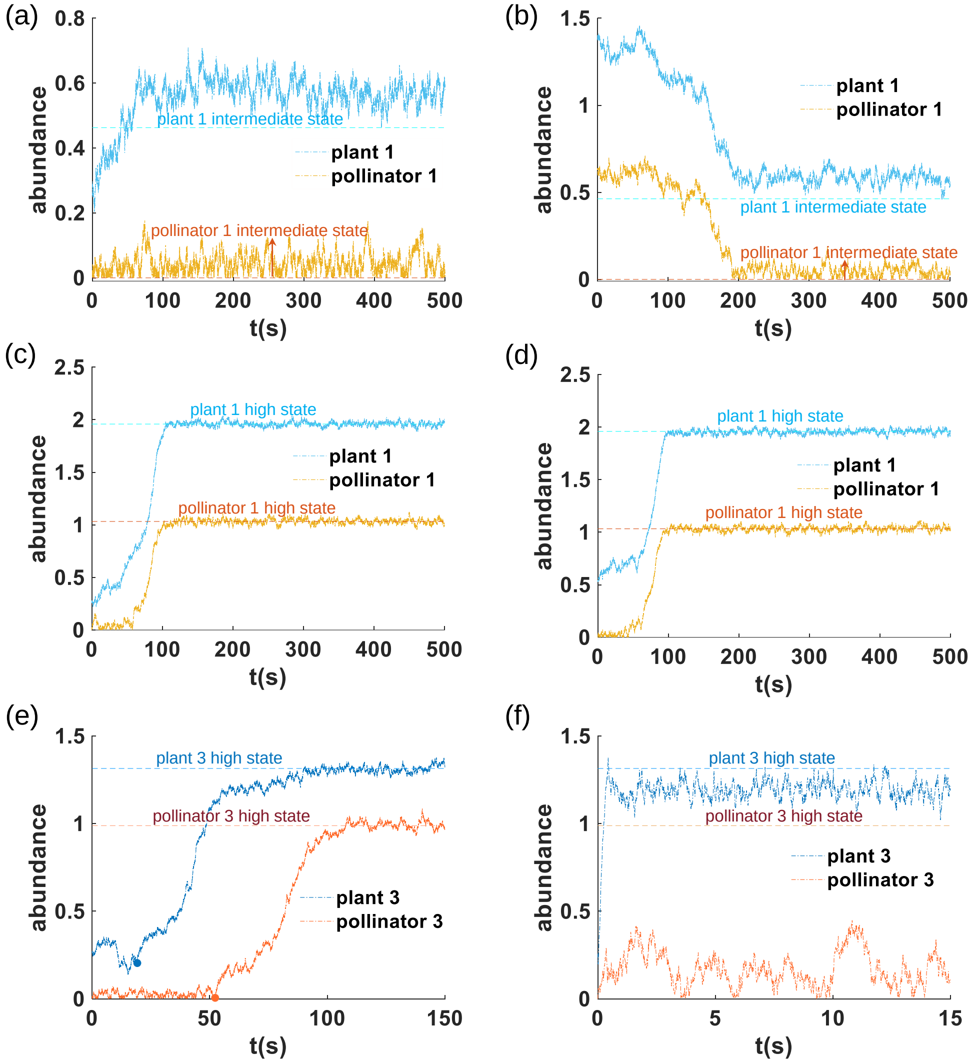

Fig A. In the case of fixed parameters, noise-induced transitions between stable states. (a-b) Taking $\kappa = 1.12$, with initial points in the low state (a) and high state (b), noise induces the system to transit to the intermediate state, presented by plant 1 and pollinator 1. (c-d) Taking $\kappa = 1.02$, with initial points in the low state (c) and intermediate state (d), noise induces the system to transit to the high state, still presented by plant 1 and pollinator 1. (e) With initial points in the low state without external force, the plants recover earlier than the pollinators, consistent with our calculated transition paths. (f) Under conditions of external intervention (keeping pollinator 2 at high abundance), the plants still recover earlier.

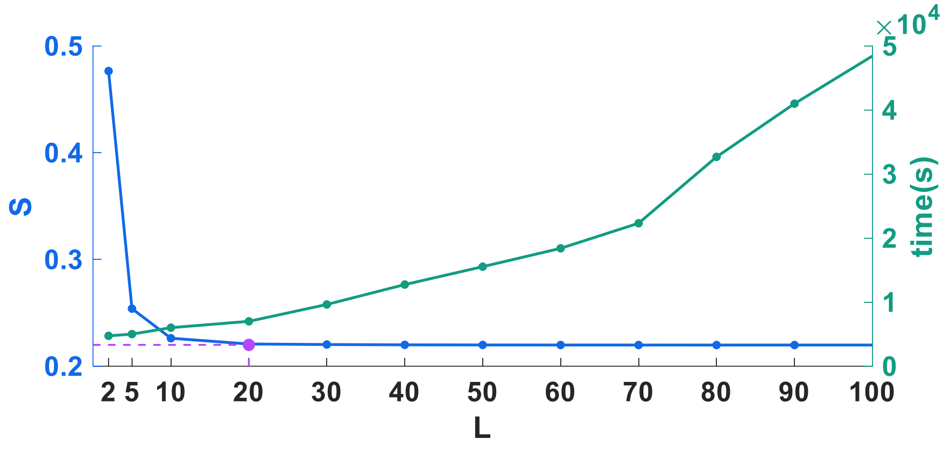

Fig B. The impact of varying L on the transition action from the high state to the low state and its computation time. Taking into account the computational considerations and the fact that the action does not significantly change when L exceeds 20, we chose an appropriate value of $L = 20$ and fixed it for subsequent analysis.

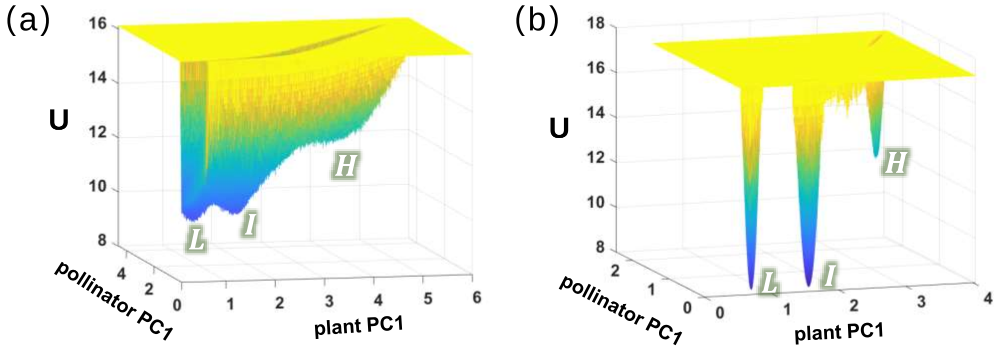

Fig C. The landscape for colored noise scenarios (red noise and blue noise). (a) The energy landscape is constructed from Langevin simulations under red noise. The system is prone to transition between stable states. (b) The energy landscape is constructed under blue noise. The transition is more difficult to occur.

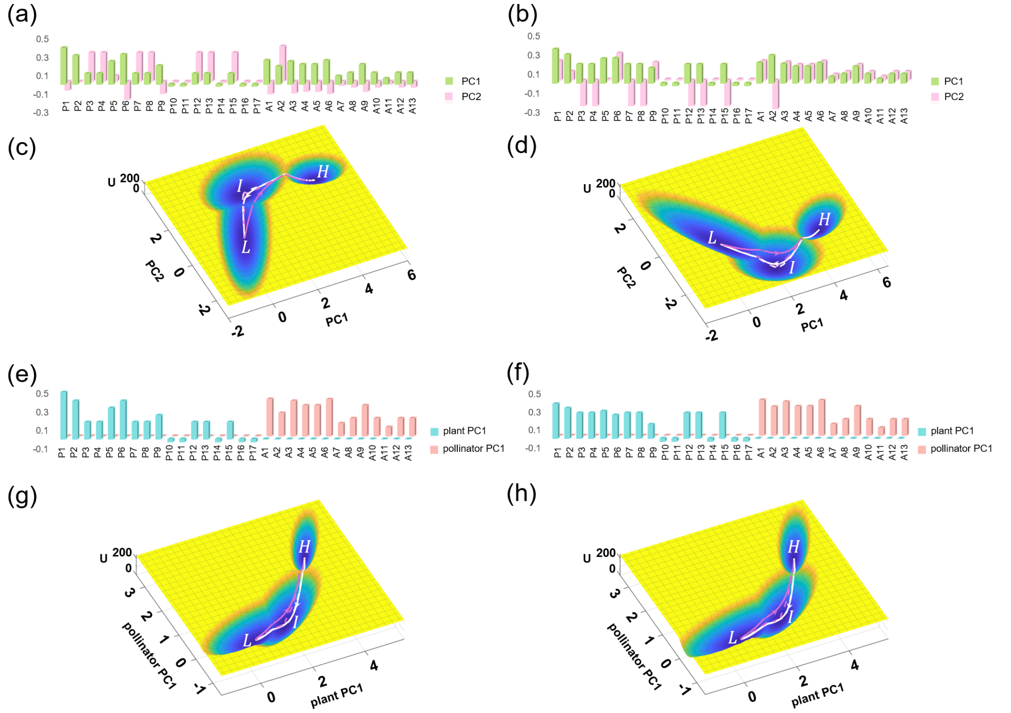

Fig D. Our generalized dimension reduction approach is more interpretable and more robust. (a and b) The weights of each plant and pollinator component in PC1 and PC2, using default parameters except κ (a: $\kappa = 1.09$, b:$\kappa = 1.1$). (c and d) The reduced landscapes corresponding to (a) and (b) respectively, using previous approach [3]. (e and f) The weights of each plant and pollinator component in plant PC1 and pollinator PC1, using default parameters except κ (e: $\kappa = 1.09$, f: $\kappa = 1.1$). (g and h) The reduced landscapes corresponding to (e) and (f) respectively, using our generalized approach.

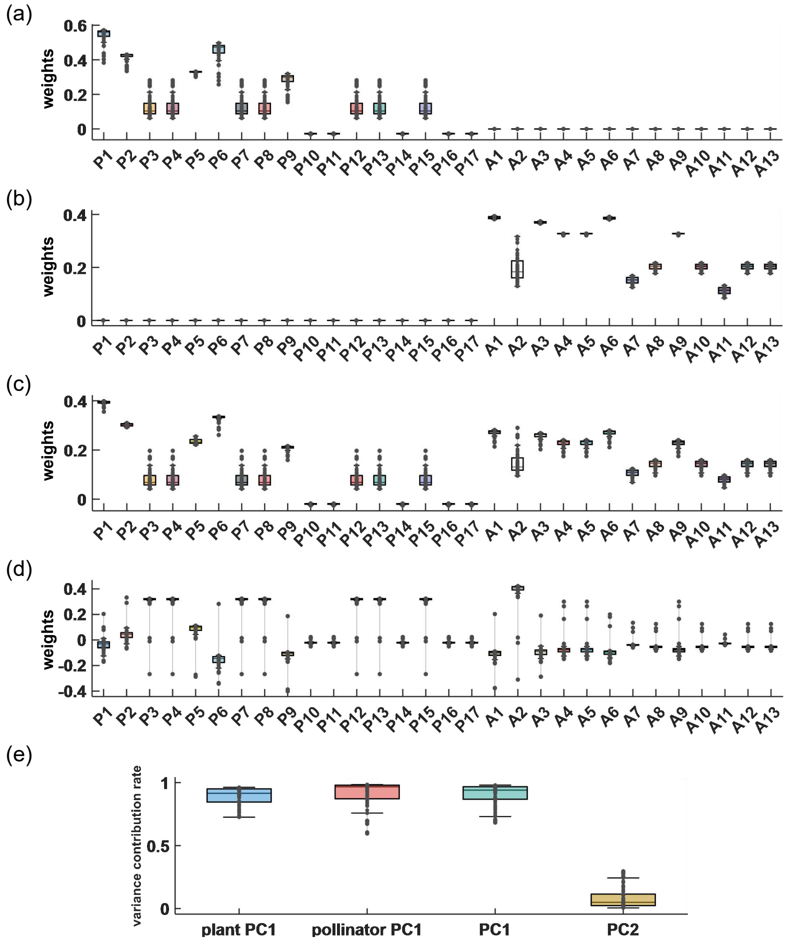

Fig E. The superiority of our approach is further illustrated by the variation of coordinate weights and variance contribution rates. (a - d) We vary different parameters and observe changes in the weights of each species that make up the coordinates: (a) plant PC1 and (b) pollinator PC1 in our approach; (c) PC1 and (d) PC2 in previous approach [3]. We use the same perturbations of parameters to ensure that two approaches are comparable. (e) The distribution of variance contribution rate of plant PC1, pollinator PC1, PC1 and PC2 With parameters varying.

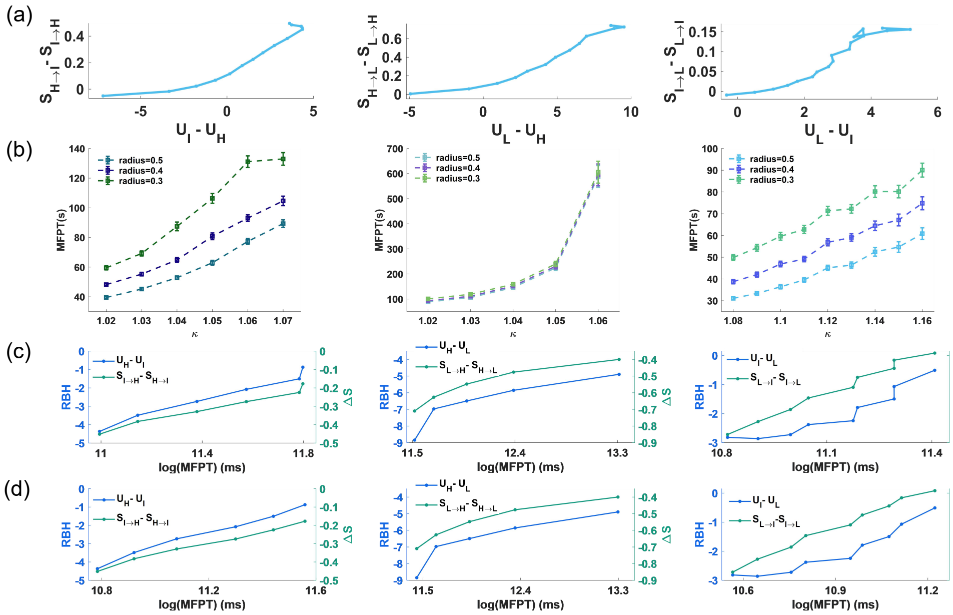

Fig F. The consistency among the barrier height, transition action as well as MFPT, which does not depend on the selection of the hyperparameter r (left: intermediate and high states, middle: low and high states, right: low and intermediate states). (a) ΔS and RBH exhibit the same trend. (b) The line graphs of MFPT from one attractor to another with varying κ, and different radii r are selected as 0.3, 0.4 and 0.5, where the error bars correspond to stand error. (c-d) Approximate linear relationships between RBH and log(MFPT) (blue line), as well as between ΔS and log(MFPT) (green line) under different r (c: $r = 0.3$, d: $r = 0.4$).

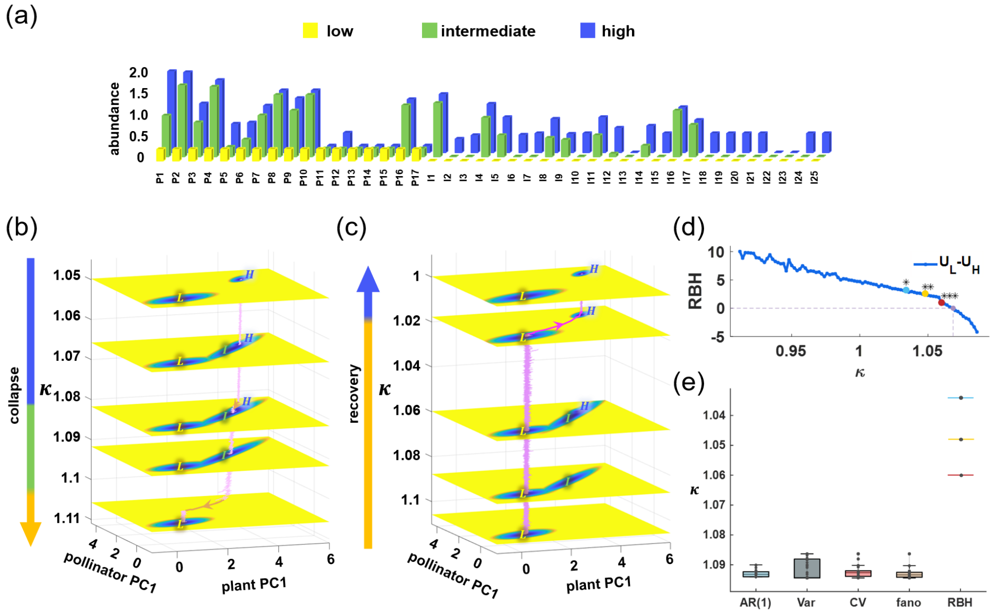

Fig G. The analysis is repeated for Network A, reproducing the superiority of RBH as an EWS. (a) The abundance of 17 plant species and 25 pollinator species (indicated by each column, see Table C for details) in low, intermediate and high stable states using the default parameters. The default values are also set as $\alpha_i^{(P)} = \alpha_j^{(A)} = 0.3, \beta_{ii}^{(P)} = \beta_{jj}^{(A)} = 1, \beta_{ik}^{(P)} = \beta_{jm}^{(A)} = 0.01 (i \neq k, j \neq m), \kappa = 1.07, \gamma_0 = 1, \delta = 0.5, h = 0.2, \mu^{(P)} = \mu^{(A)} = 0.001$. (b-c) The process of system collapse (b) and recovery (c) is simulated from the Langevin equations, by modelling linear change in κ. The system goes through the intermediate state for the collapse, while directly transits to the high state for the recovery. (d) Calculated RBH between low and high states with increasing κ. Three marked points are with the same meaning as the main text. (e) For complete collapse, the RBH again serves as the earliest warning signal compared with AR(1), variance (Var), coefficient of variation (CV) and fano factor (fano).

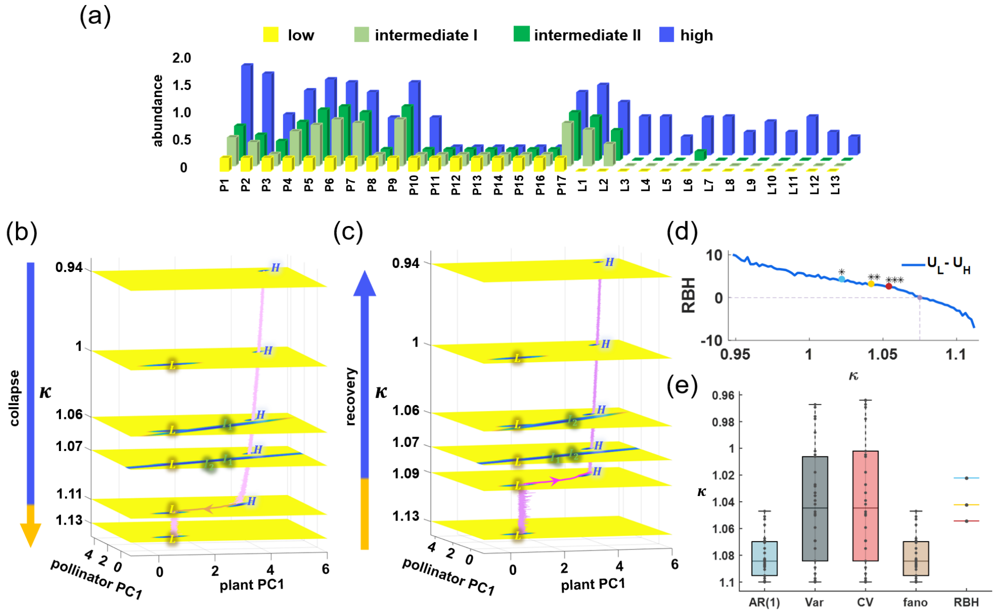

Fig H. The analysis is repeated for Network C, reproducing the superiority of RBH as an EWS. (a) The abundance of 17 plant species and 13 pollinator species (indicated by each column, see Table E for details) in low, intermediate and high stable states using the same default parameters. (b-c) The process of system collapse (b) and recovery (c) is simulated from the Langevin equations. (d) Calculated RBH between low and high states with increasing κ. Three marked points are with the same meaning as the main text and Fig Gd. (e) For complete collapse, if we choose $p < 0.05$ (blue line) or $p < 0.01$ (gold line), the RBH can serve as the earliest warning signal compared with AR(1), variance (Var), coefficient of variation (CV) and fano factor (fano).

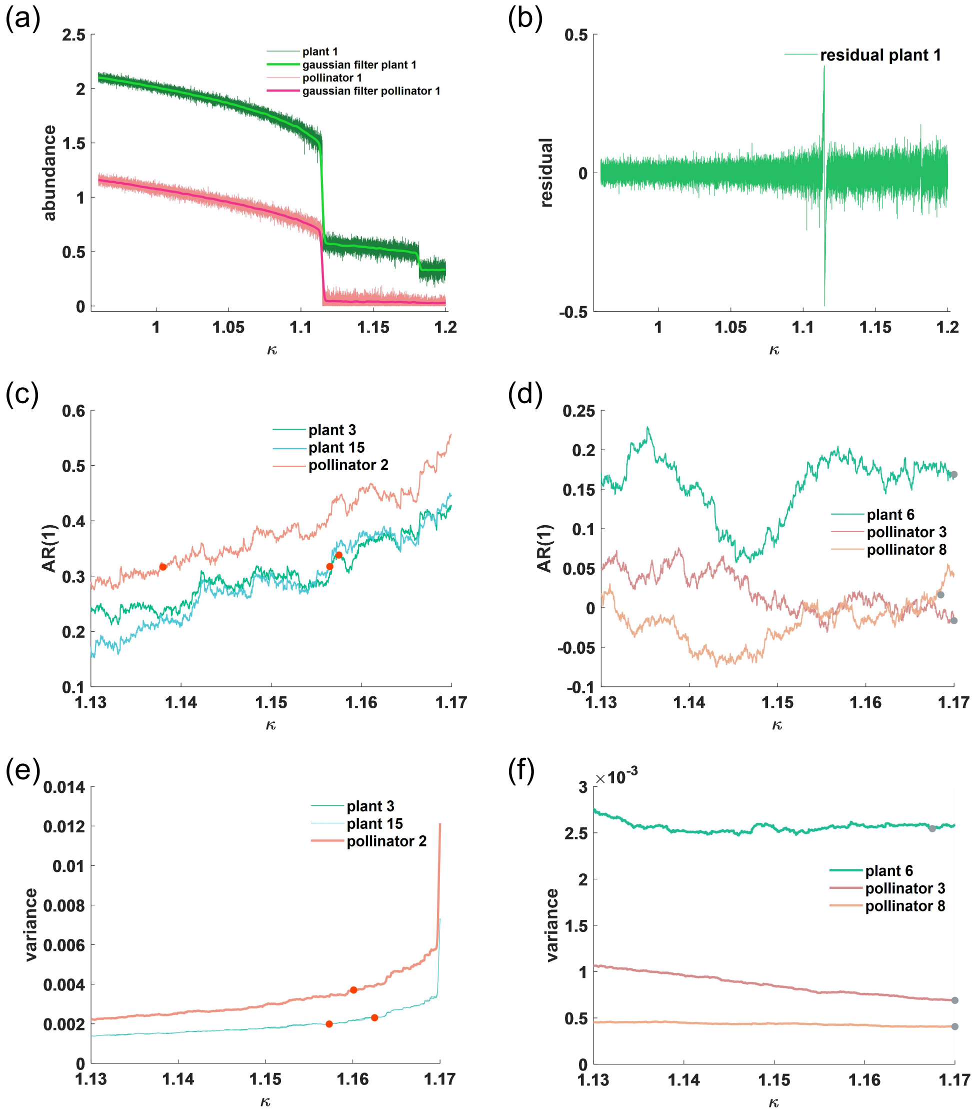

Fig I. Interpretation of the time-series metrics. (a) Species abundance fluctuations and shifts are shown under the parameter κ increasing. Gaussian filtering algorithms are used for preprocessing, presented by plant 1 and pollinator 1. (b) The residual is obtained from the difference between the original abundance and the Gaussian filtering results, presented by plant 1. Traditional time-series indicators rely on the residuals to determine the occurrence of critical points, which is identified for the continued increase of the indicators. (c-f) We examine whether all species can achieve early warning. Some species can, like plant 3, plant 15 and pollinator 2 (red points in c and e); while some species cannot, like plant 6, pollinator 3 and pollinator 8 (grey points in d and f).

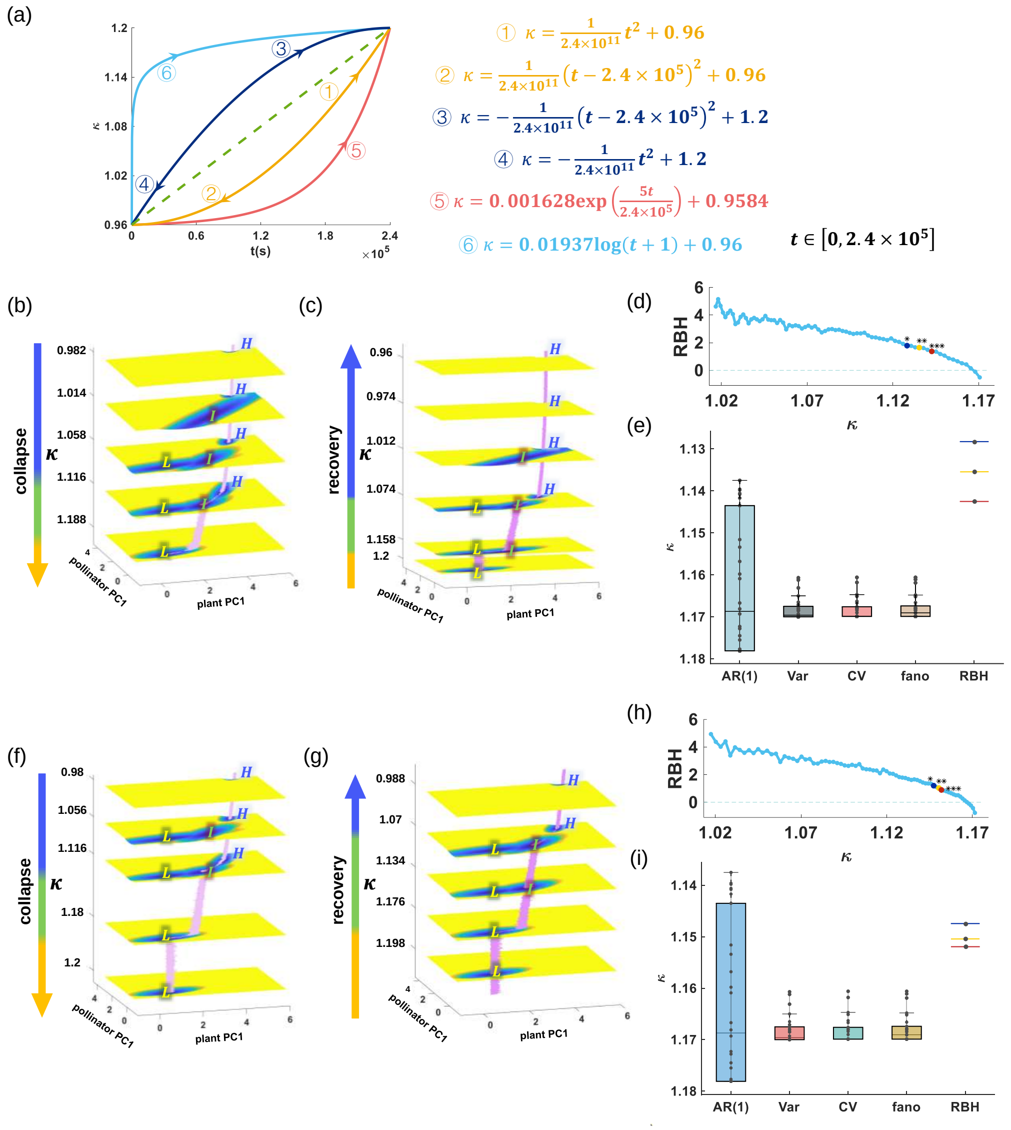

Fig J. By simulating the nonlinear changes in κ, we again demonstrate the effectiveness of RBH as an EWS. (a) We simulate κ changing over time in quadratic increase (① and ③), quadratic decrease (② and ④), exponential increase (⑤), and logarithmic increase (⑥). (b-c) Corresponding to ① and ②, the recovery requires better conditions (smaller κ) than the collapse. (d-e) We show the RBH at the scenario of ① (d) and its best predictive effectiveness compared with traditional indicators (e). (f-g) Corresponding to ③ and ④, the recovery also requires better conditions (smaller κ) than the collapse. (h-i) We show the RBH at the scenario of ③ (h) and RBH once again serves as the earliest warning signal (i).

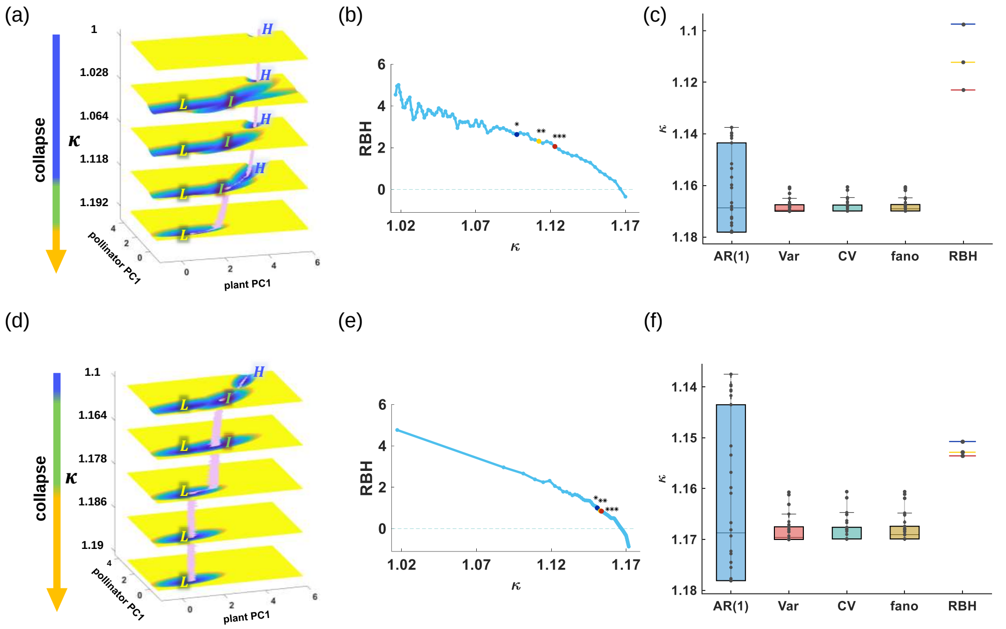

Fig K. The scenarios of exponential and logarithmic changes in κ. (a) Corresponding to ⑤ in Fig Ja (the exponential changes in κ), (b-c) we showed the RBH variation (b) and identified its earliest warning (c), which implies that the rapid change contributes to achieving earlier warnings. (d) Corresponding to ⑥ in Fig Ja (the logarithmic changes in κ), (e-f) we showed the RBH variation (e) and identified that RBH's warning occurs later than the exponential scenario, but it is still superior to the traditional indicators on average (f).

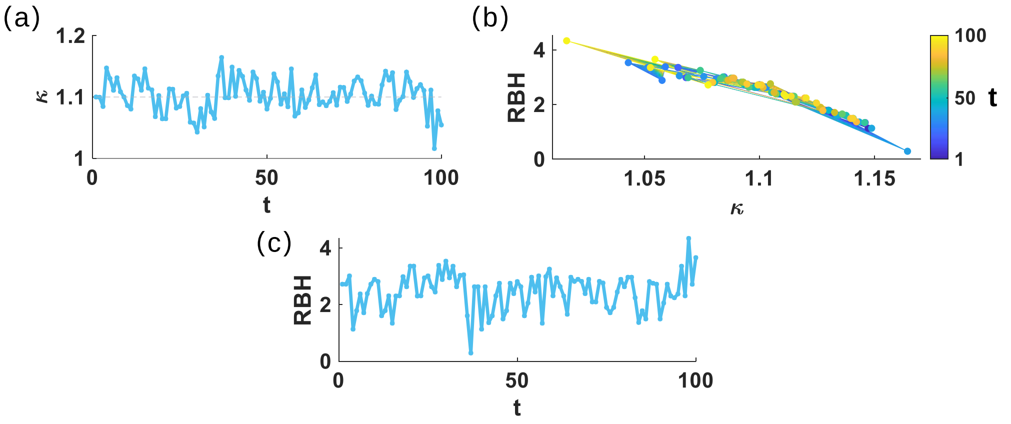

Fig L. To show the RBH's inability to predict transitions that have not occurred, we perturbed κ in an autocorrelated manner. (a) We simulate κ fluctuating over time with weak autocorrelation. (b) The corresponding RBH varies with changes in κ, and the color bar represents the time progression. (c) The RBH evolves over time, and there is no statistically significant point in the sequence to reject the BDS test.

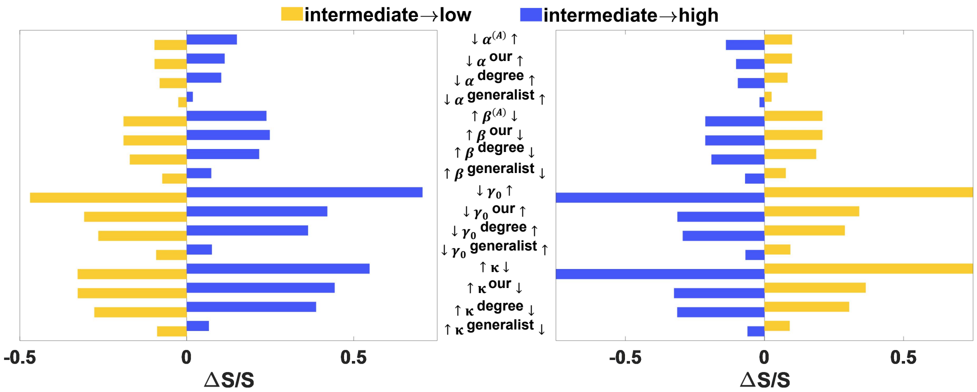

Fig M. Global sensitivity analysis is repeated for Network A, to emphasize the indispensable role of pollinators surviving in the intermediate state. We perturbed 1 % of each parameter from the default value to guarantee the existence of the intermediate state. The left panel corresponds to the perturbations in the direction of collapse, and the right corresponds to the opposite perturbations. We reported the relative changes in transition actions of intermediate to low transition and intermediate to high transition. No superscript ($\alpha^{(A)}, \beta^{(A)}, \gamma_0, \kappa$) means perturbation on all species parameters. The superscript 'our' means only perturbation on the parameters of the pollinator species surviving in the intermediate state (I1, I4, I5, I8, I9, I11, I12, I14, I16, I17). The superscript 'degree' means only perturbation on the parameters of the same number of pollinator species with larger degrees (I1, I2, I3, I4, I5, I6, I8, I11, I12, I16). The superscript 'generalist' means only perturbation on the parameters of the most generalist pollinator species (I1). Increasing γ_0 or decreasing κ makes the relative change be -1 and infinity, and for visualization, we denoted them as -0.75 and 0.75 respectively.

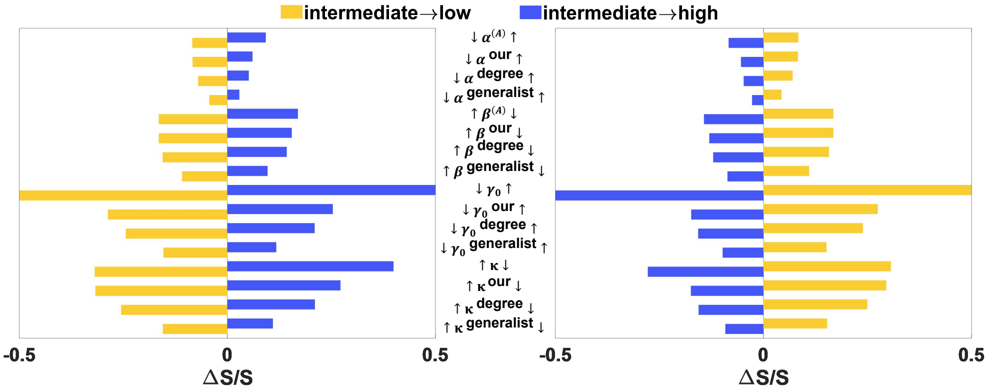

Fig N. Global sensitivity analysis is repeated for Network C, to emphasize the indispensable role of pollinators surviving in the intermediate state. We perturbed 0.5 % of each parameter from the default value, and the presentation follows Fig M. The superscript 'our' means only perturbation on the parameters of the pollinator species surviving in the intermediate state (L1, L2, L6). The superscript 'degree' means only perturbation on the parameters of the same number of pollinator species with larger degrees (L1, L2, L9). The superscript 'generalist' means only perturbation on the parameters of the most generalist pollinator species (L1). Increasing or decreasing γ_0 makes the relative change be -1 and infinity, and for visualization, we denoted them as -0.5 and 0.5 respectively.

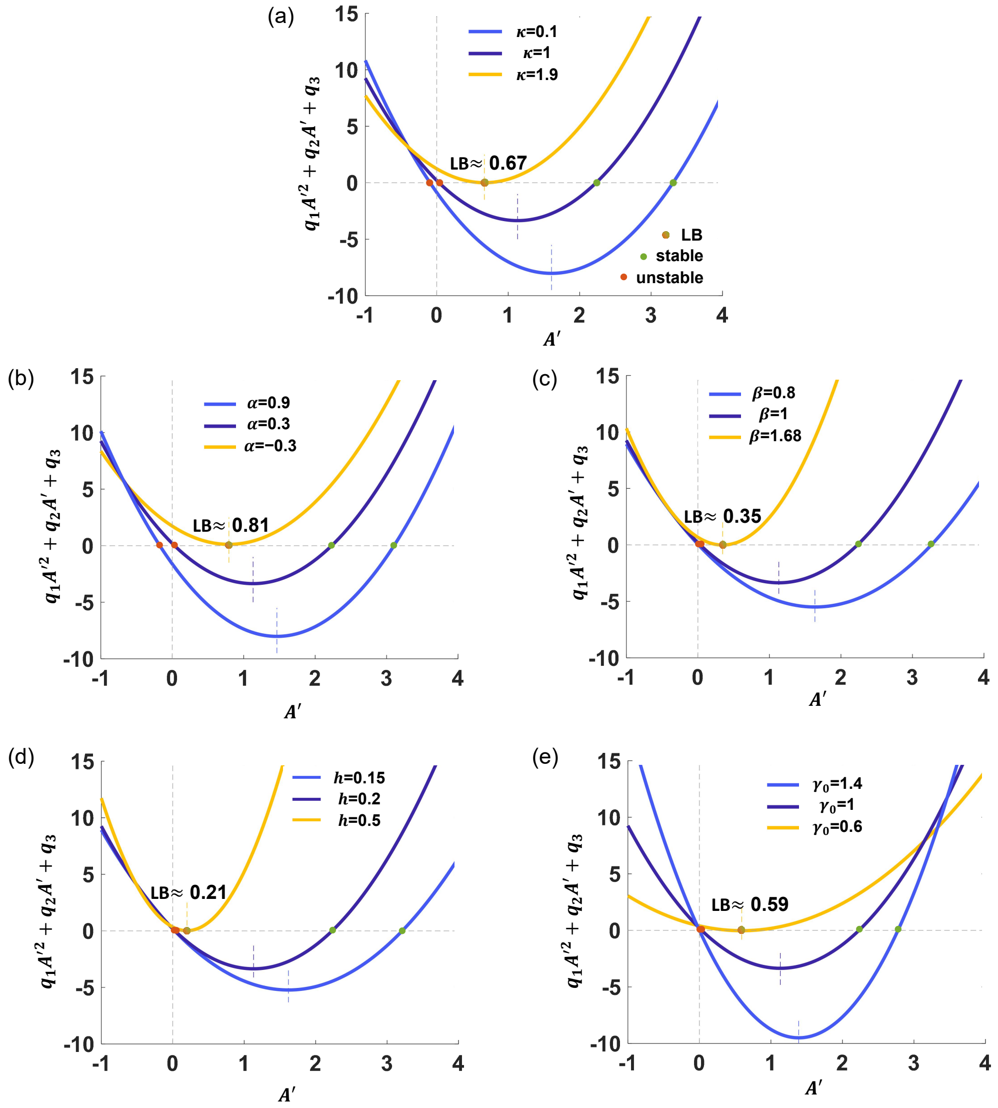

Fig O. For the two-dimensional reduced model, the numerical simulation results of perturbation on other parameters, consistent with our theoretical analysis (Table B). The colored dashed line represents the axis of symmetry for the quadratic function. Since the effect induced by parameter changes is monotonic, we show only three cases: (i) The dark blue line represents the baseline using the default parameters. (ii) The blue line indicates better conditions, including lower κ, β, h and larger α, γ_0. (iii) The gold line gives the solution corresponding to the smallest stable point (lower bound). (a) We increase only κ and fix other parameters. The stable point (larger root) gradually becomes smaller until it approaches the lower bound (LB). (b)-(e) We fix $\kappa = 1$ and change individual parameter including α (b), β (c), h (d), and γ_0 (e). The variations in A_5 LB, the axis of symmetry, $A_4 * A_5$ and A_5 are also consistent with our derived theoretical results (Table B). The default values are set as follows: $\alpha = 0.3$, $\beta = 1$, $h = 0.2$, $\gamma_0 = 1$ and $\kappa = 1$.

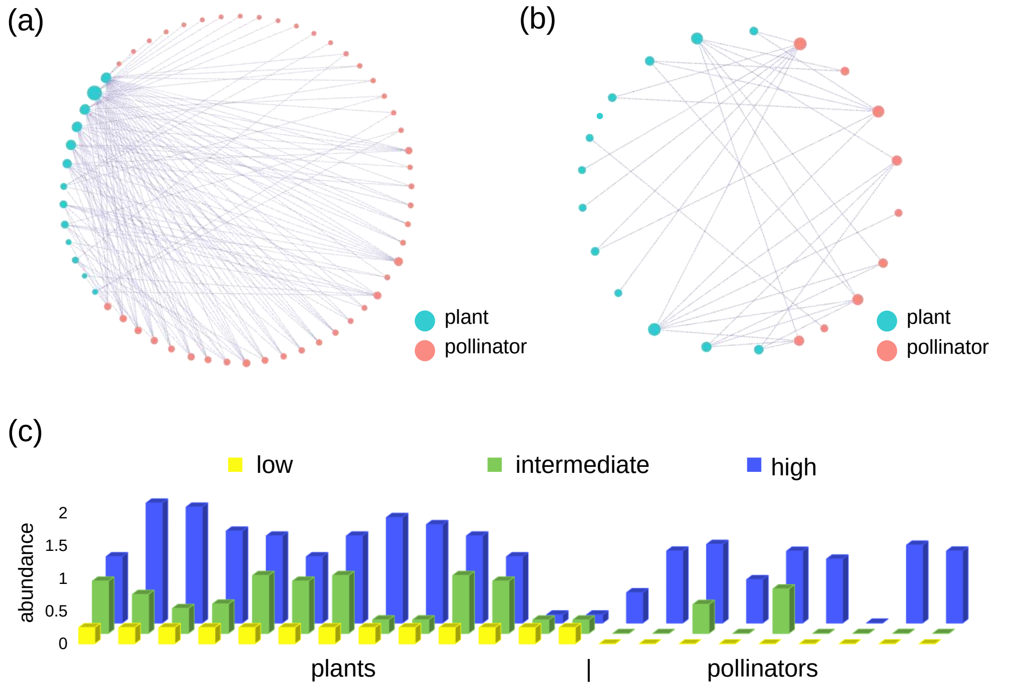

Fig P. A new network from the Web-of-Life database to reproduce our results. (a) The network is derived from Motten in 1986, which comprises 44 pollinators, 13 plants, and 143 interactions. (b) After removing pollinators at random, a subnetwork exhibiting tristability is randomly selected, which includes 9 pollinators and 13 plants (Network B, Table D). (c) The abundance of all species is shown at default parameters in high state, intermediate state, and low state. The default parameter values are set as follows: $\alpha_i^{(P)} = \alpha_j^{(A)} = 0.3$, $\beta_{ik}^{(P)} = \beta_{jm}^{(A)} = 0.01 (i \neq k, j \neq m)$, $\beta_{ii}^{(P)} = \beta_{jj}^{(A)} = 1$, $\kappa = 1.1$, $\gamma_0 = 1$, $\delta = 0.5$, $h = 0.2$, $\mu^{(P)} = \mu^{(A)} = 0.001$.

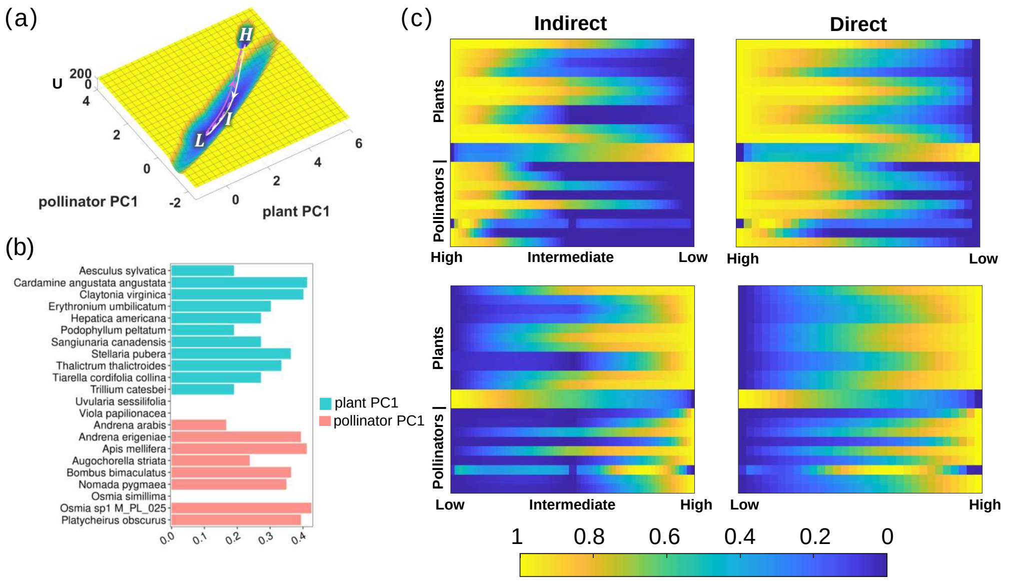

Fig Q. The transition path indicates the significant role of the intermediate state. (a) The reduced-dimensional tristable landscape under default parameters. (b) The weights of each plant and pollinator component in plant PC1 and pollinator PC1. (c) The multidimensional transition paths between attractors, which again suggest that the system tends to go through the intermediate state at the transition from the high state to the low state.

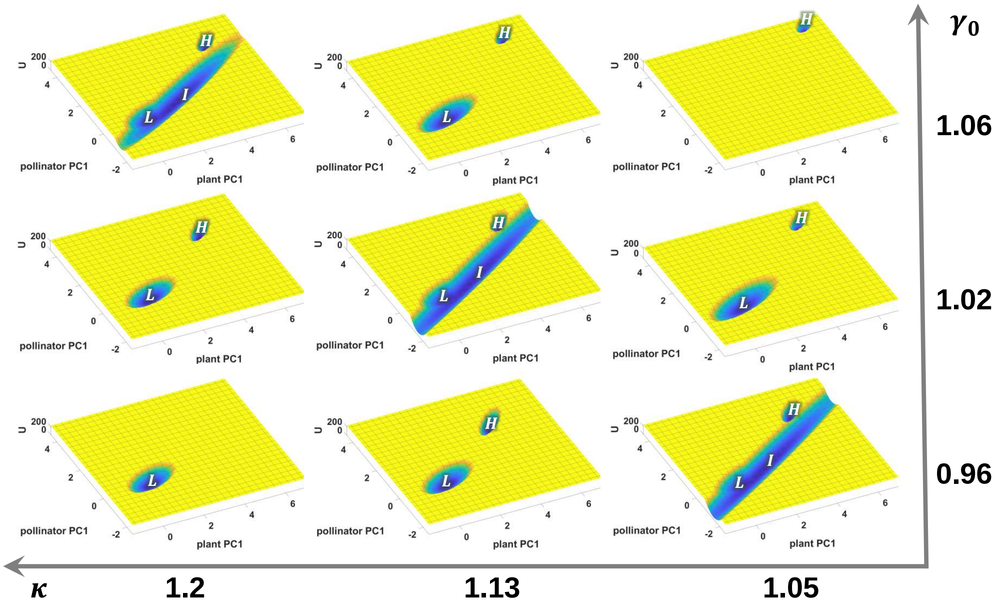

Fig R. The landscape changes with parameters κ and γ_0 variation, showing different stable states. The projection coordinates are all computed under the conditions of $\kappa = 1.1$ and $\gamma_0 = 1$.

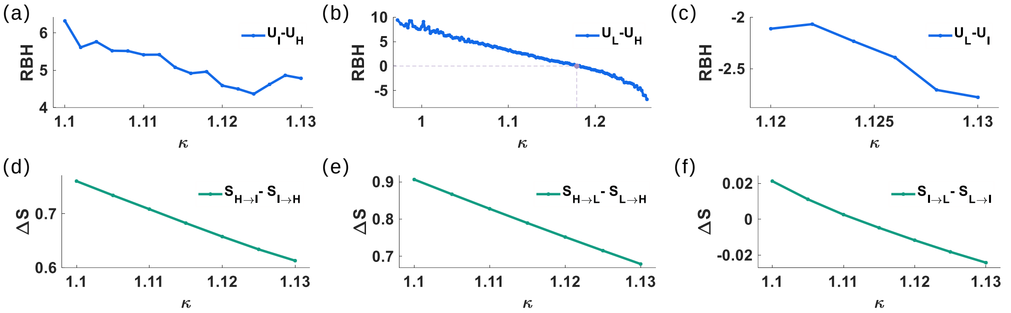

Fig S. Consistency between barrier height and transition action. (a-c) Line graphs of RBH between stable-state pairs with varying κ (a: intermediate and high states; b: low and high states; c: low and intermediate states). (d-f) The difference for the minimum actions between the forward and backward transitions (ΔS) vary with κ (d: intermediate and high state; e: low and high state; f: low and intermediate state).

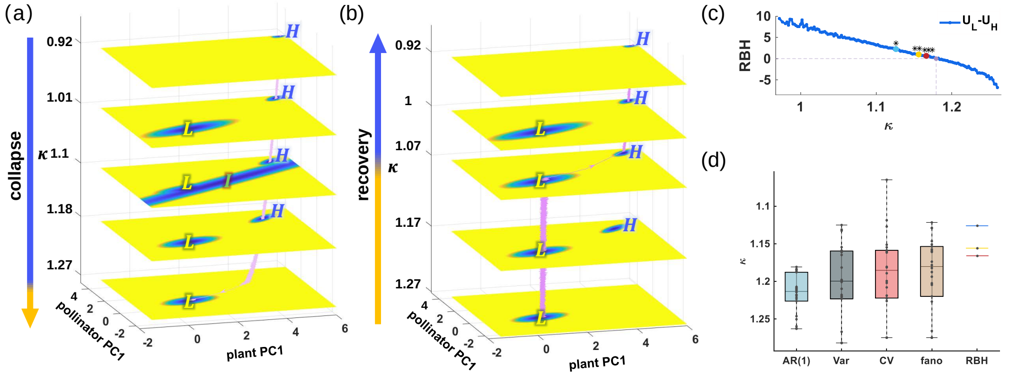

Fig T. The analysis is repeated for Network B, reproducing the superiority of RBH as an EWS. (a-b) The process of system collapse (a) and recovery (b) is simulated from the Langevin equations. (c) Calculated RBH between low and high states with increasing κ. Three marked points are with the same meaning as the main text and Figs Gd and Hd. (d) For complete collapse, on average, the RBH can serve as the earliest warning signal compared with other time-series metrics.

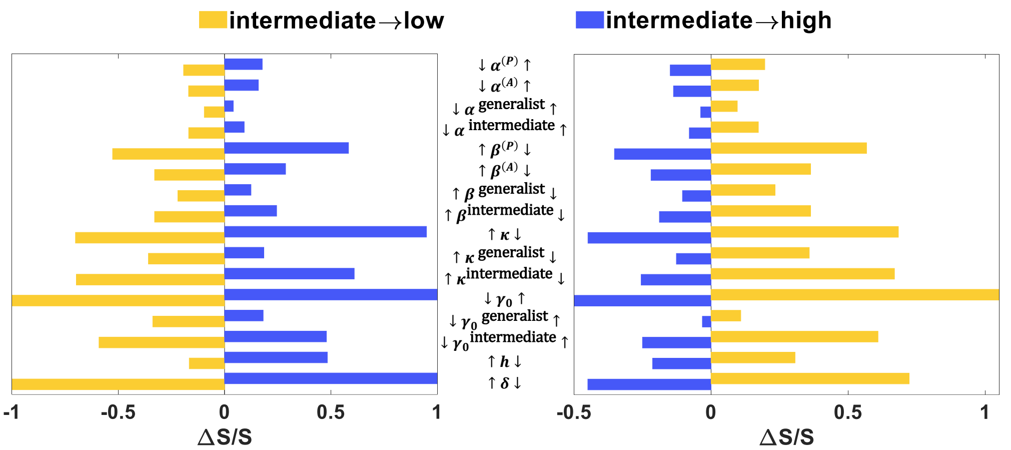

Fig U. Global sensitivity analysis is repeated for Network B, to emphasize the indispensable role of pollinators surviving in the intermediate state. We perturbed 1 % of each parameter from the default value, and the presentation follows Figs M and N. No superscript ($\alpha^{(A)}, \beta^{(A)}, \gamma_0, \kappa$,$h$,$\delta$) means perturbation on all species parameters. The superscript 'generalist' means only perturbation on the parameters of the most generalist pollinator species (Q5). The superscript 'intermediate' means only perturbation on the parameters of the pollinator species surviving in the intermediate state (Q3, Q5). Perturbing γ_0 and δ makes the relative change be -1 or infinity, and for visualization, we denoted them as -1 and 1 respectively.

Table A. The plant-pollinator network exhibiting tristability in our text

	A1	A2	A3	A4	A5	A6	A7	A8	A9	A10	A11	A12	A13
P1	192	3	17	19	14	5	0	6	2	1	0	1	1
P2	0	47	0	1	1	4	9	0	3	0	0	0	0
P3	0	18	0	0	0	0	0	0	0	0	0	0	0
P4	0	4	0	0	0	0	0	0	0	0	0	0	0
P5	6	2	3	0	0	0	0	0	0	0	1	0	0
P6	2	0	16	0	0	1	0	0	0	0	0	0	0
P7	0	1	0	0	0	0	0	0	0	0	0	0	0
P8	0	2	0	0	0	0	0	0	0	0	0	0	0
P9	2	0	0	0	0	0	0	0	0	0	0	0	0
P10	0	0	0	0	0	0	0	0	0	0	0	0	0
P11	0	0	0	0	0	0	0	0	0	0	0	0	0
P12	0	2	0	0	0	0	0	0	0	0	0	0	0
P13	0	3	0	0	0	0	0	0	0	0	0	0	0
P14	0	0	0	0	0	0	0	0	0	0	0	0	0
P15	0	2	0	0	0	0	0	0	0	0	0	0	0
P16	0	0	0	0	0	0	0	0	0	0	0	0	0
P17	0	0	0	0	0	0	0	0	0	0	0	0	0

Index	P1	P2	P3	P4	P5
Species	Leucanthemum vulgare	Lotus corniculatus	Lathyrus pratensis	Vicia cracca	Hypochoeris radicata
Index	P6	P7	P8	P9	P10
Species	Ranunculus acris	Trifolium dubium	Prunella vulgaris	Achillea millefolium	Centaurea nigra
Index	P11	P12	P13	P14	P15
Species	Trifolium repens	Vicia sativa	Lychnis flos-cuculi	Plantago lanceolata	Trifolium pratense
Index	P16	P17			
Species	Cirsium arvense	Taraxacum officinale			

Index	A1	A2	A3	A4	A5
Species	Eristalinus sepulcharius	Bombus pascuorum	Anthomyidae sp1 M_PL_006	Eristalis tenax	Scathophaga stercoraria
Index	A6	A7	A8	A9	A10
Species	Lasiommata megera	Megachile willughbiella	Micromoth sp1 M_PL_006	Polyommatus icarus	Adela rufimatrella
Index	A11	A12	A13		
Species	Lasioglossum leucozonium	Nemotelus pantherinus	Oligia sp1 M_PL_006		

Table B. Monotonic effect by parameters changed in one direction

	κ_1	A_5 lower bound	the axis of symmetry	$A_4 * A_5$	A_5
$\kappa\uparrow$	o	o	$-$	$+$	$-$
$\alpha\uparrow$	$+$	$-$	$+$	$-$	$+$
$\beta\uparrow$	$-$	$-$	$-$	$+$	$-$
$h\uparrow$	$-$	$-$	$-$	$/$	$-$
$\gamma_0\uparrow$	$+$	$+$	$+$	$-$	$+$

Table C. Network A: more pollinators surviving in the intermediate state

	I1	I2	I3	I4	I5	I6	I7	I8	I9	I10	I11	I12	13	I14	I15	I16	I17	I18	I19	I20	I21	I22	I23	I24	I25
P1	19	55	35	5	19	5	15	5	0	8	7	4	0	4	5	0	0	2	2	1	1	0	0	1	1
P2	19	0	0	7	1	0	0	4	9	0	1	0	0	0	0	2	2	0	0	0	0	0	0	0	0
P3	0	0	0	1	0	0	0	0	0	0	0	1	0	0	0	0	0	0	0	0	0	0	0	0	0
P4	7	0	0	4	0	0	0	0	0	0	0	0	0	0	0	1	1	0	0	0	0	0	0	0	0
P5	0	0	1	0	0	0	0	0	0	0	0	1	0	0	0	0	0	0	0	0	0	1	0	0	0
P6	0	0	0	0	0	11	0	1	0	0	0	0	5	0	0	0	0	0	0	0	0	0	1	0	0
P7	0	0	0	8	0	0	0	0	0	0	0	0	0	0	0	0	0	0	0	0	0	0	0	0	0
P8	9	0	0	0	0	0	0	0	0	0	0	0	0	0	0	1	0	0	0	0	0	0	0	0	0
P9	3	0	0	0	0	0	0	0	0	0	0	0	0	1	0	0	0	0	0	0	0	0	0	0	0
P10	5	0	0	0	0	0	0	0	0	0	0	0	0	0	0	1	0	0	0	0	0	0	0	0	0
P11	0	0	0	0	0	0	0	0	0	0	0	0	0	0	0	0	0	0	0	0	0	0	0	0	0
P12	0	1	0	0	0	0	0	0	0	0	0	0	0	0	0	0	0	0	0	0	0	0	0	0	0
P13	0	0	0	0	0	0	0	0	0	0	0	0	0	0	0	0	0	0	0	0	0	0	0	0	0
P14	0	0	0	0	0	0	0	0	0	0	0	0	0	0	0	0	0	0	0	0	0	0	0	0	0
P15	0	0	0	0	0	0	0	0	0	0	0	0	0	0	0	0	0	0	0	0	0	0	0	0	0
P16	1	0	0	0	0	0	0	0	0	0	0	0	0	0	0	0	0	0	0	0	0	0	0	0	0
P17	0	0	0	0	0	0	0	0	0	0	0	0	0	0	0	0	0	0	0	0	0	0	0	0	0

Index	I1	I2	I3	I4	I5
Species	Maniola jurtina	Pollenia sp1 M_PL_006	Eristalis nemorum	Andrena wilkella	Eristalis tenax
Index	I6	I7	I8	I9	I10
Species	Cheilosia albitarsis	Empis livida	Lasiommata megera	Megachile willughbiella	Lycaena phlaeas
Index	I11	I12	I13	I14	I15
Species	Odontomyia tigrina	Sphaerophoria sp1 M_PL_006	Neoascia tenur	Odontomyia viridula	Rhagio tringarius
Index	I16	I17	I18	I19	I20
Species	Zygaena filipendulae	Ochlodes venata	Eriothrix rufomaculatus	Eristalis pertinax	Adela rufimatrella
Index	I21	I22	I23	I24	I25
Species	Anosymia sp1 M_PL_006	Lasioglossum leucozonium	Lasioglossum villosulum	Nemotelus pantherinus	Tetanocera ferruginea

Table D. Network B: 13 plants and 9 pollinators with tristbility

	Q1	Q2	Q3	Q4	Q5	Q6	Q7	Q8	Q9
H1	0	0	0	0	1	0	0	0	0
H2	1	1	1	0	1	1	0	1	1
H3	0	1	1	1	0	1	0	1	1
H4	0	0	1	1	0	0	0	1	0
H5	0	0	1	0	1	0	0	0	0
H6	0	0	0	0	1	0	0	0	0
H7	0	0	1	0	1	0	0	0	0
H8	0	1	0	0	0	1	0	1	1
H9	0	1	0	0	0	0	0	1	1
H10	0	0	1	0	1	0	0	0	0
H11	0	0	0	0	1	0	0	0	0
H12	0	0	0	0	0	0	0	0	0
H13	0	0	0	0	0	0	1	0	0

Index	H1	H2	H3	H4	
Species	Aesculus sylvatica	Cardamine angustata	Claytonia virginica	Erythronium umbilicatum	
Index	H5	H6	H7	H8	
Species	Hepatica americana	Podophyllum peltatum	Sangiunaria canadensis	Stellaria pubera	
Index	H9	H10	H11	H12	H13
Species	Thalictrum thalictroides	Tiarella cordifolia collina	Trillium catesbei	Uvularia sessilifolia	Viola papilionacea

Index	Q1	Q2	Q3	Q4	
Species	Andrena arabis	Andrena erigeniae	Apis mellifera	Augochorella striata	
Index	Q5	Q6	Q7	Q8	Q9
Species	Bombus bimaculatus	Nomada pygmaea	Osmia simillima	Osmia sp1 M_PL_025	Platycheirus obscurus

Table E. Network C: tetrastbility including two intermediate states

	L1	L2	L3	L4	L5	L6	L7	L8	L9	L10	L11	L12	L13
P1	225	192	68	10	0	4	2	5	0	3	1	2	0
P2	8	0	1	1	9	0	3	0	2	0	1	0	1
P3	0	0	0	0	0	1	0	0	0	0	0	0	0
P4	1	0	0	0	0	0	0	0	1	0	0	0	0
P5	4	6	0	0	0	1	0	0	0	0	0	0	0
P6	1	2	0	0	0	0	0	0	0	0	0	0	0
P7	1	0	0	0	0	0	0	0	0	0	0	0	0
P8	0	0	0	0	0	0	0	0	1	0	0	0	0
P9	1	2	0	0	0	0	0	0	0	0	0	0	0
P10	0	0	0	0	0	0	0	0	1	0	0	0	0
P11	0	0	0	0	0	0	0	0	0	0	0	0	0
P12	0	0	0	0	0	0	0	0	0	0	0	0	0
P13	0	0	0	0	0	0	0	0	0	0	0	0	0
P14	0	0	0	0	0	0	0	0	0	0	0	0	0
P15	0	0	0	0	0	0	0	0	0	0	0	0	0
P16	0	0	0	0	0	0	0	0	0	0	0	0	0
P17	1	0	0	0	0	0	0	0	0	0	0	0	0

Index	L1	L2	L3	L4
Species	Helophilus sp1 M_PL_006	Eristalinus sepulcharius	Lucillia sp1 M_PL_006	Psithyrus vest
Index	L5	L6	L7	L8
Species	Megachile willughbiella	Sphaerophoria sp1 M_PL_006	Polyommatus icarus	Rhagio tringarius
Index	L9	L10	L11	L12
Species	Zygaena filipendulae	Melanostoma sp1 M_PL_006	Andrena pubescens	Eristalis pertinax
Index	L13			
Species	Pyronia tithonus			
